# Recommendations for analgesia and sedation in critically ill children admitted to intensive care unit

**DOI:** 10.1186/s44158-022-00036-9

**Published:** 2022-02-12

**Authors:** Angela Amigoni, Giorgio Conti, Alessandra Conio, Manuela Corno, Paola Claudia Fazio, Federica Ferrero, Marta Gentili, Cristina Giugni, Manuela L’Erario, Maristella Masola, Paola Moliterni, Giuseppe Pagano, Zaccaria Ricci, Stefano Romagnoli, Beatrice Vasile, Francesca Vitale, Geremia Zito Marinosci, Maria Cristina Mondardini

**Affiliations:** 1 University Hospital, Via Giustiniani 3, 35128 Padova, Italy; 2 Catholic A. Gemelli University Hospital Roma, Rome, Italy; 3 Regina Margherita Hospital, Torino, Italy; 4 ASST Pope Giovanni XIII Hospital, Bergamo, Italy; 5 Maggiore Hospital, Novara, Italy; 6 Pharmacovigilance Service, Unit of Clinical Pharmacology, Department of Biomedical and Clinical Sciences L. Sacco, “Luigi Sacco” University Hospital, ASST Fatebenefratelli-Sacco, Milan, Italy; 7 Pharmacy Unit, San Gerardo Hospital, Monza, Italy; 8 Meyer Hospital, Firenze, Italy; 9 Pope Giovanni XIII Pediatric Hospital, Bari, Italy; 10 University Hospital, Verona, Italy; 11 Careggi University-Hospital, Firenze, Italy; 12 Spedali Civili Hospital, Brescia, Italy; 13 Ospedale Santobono Hospital, Naples, Italy; 14 University Hospital IRCCS S.Orsola, Bologna, Italy

**Keywords:** Sedation, Paediatric intensive care unit, Pain, Substance withdrawal syndrome, Delirium

## Abstract

We aim to develop evidence-based recommendations for intensivists caring for children admitted to intensive care units and requiring analgesia and sedation. A panel of national paediatric intensivists expert in the field of analgesia and sedation and other specialists (a paediatrician, a neuropsychiatrist, a psychologist, a neurologist, a pharmacologist, an anaesthesiologist, two critical care nurses, a methodologist) started in 2018, a 2-year process. Three meetings and one electronic-based discussion were dedicated to the development of the recommendations (presentation of the project, selection of research questions, overview of text related to the research questions, discussion of recommendations). A telematic anonymous consultation was adopted to reach the final agreement on recommendations. A formal conflict-of-interest declaration was obtained from all the authors. Eight areas of direct interest and one additional topic were considered to identify the best available evidence and to develop the recommendations using the Evidence-to-Decision framework according to the Grading of Recommendations Assessment, Development and Evaluation (GRADE) approach. For each recommendation, the level of evidence, the strength of the recommendation, the benefits, the harms and the risks, the benefit/harm balance, the intentional vagueness, the values judgement, the exclusions, the difference of the opinions, the knowledge gaps, and the research opportunities were reported. The panel produced 17 recommendations. Nine were evaluated as strong, 3 as moderate, and 5 as weak. Conclusion*:* a panel of national experts achieved consensus regarding recommendations for the best care in terms of analgesia and sedation in critically ill children.

**Supplementary Information:**

The online version contains supplementary material available at 10.1186/s44158-022-00036-9.

## Background

Analgesia and sedation are essential in the care of critically ill children. This is particularly true during intubation and mechanical ventilation, but controlling pain and agitation, reducing discomfort, allowing invasive procedures, and avoiding accidental removal of medical devices are other crucial goals to achieve in these patients. Furthermore, pain control promotes the reduction of oxygen demand, and this result can only be achieved using a goal-directed strategy of treatment and a validated assessment of the patient’s level of comfort. Under- and overtreatment are both harmful. Under-sedation does not allow to obtain adequate physiological and physical distress control. Conversely, over sedation may delay recovery, cause tolerance and possibly promote the development of withdrawal syndrome.

In 2006, Playfor et al. [1], for the United Kingdom Pediatric Intensive Care Society Sedation Analgesia and Neuromuscular Blockade Working Group, published consensus guidelines on analgesia and sedation in critically ill children and underlined the paucity of high-quality literature on this topic. This publication represents a milestone for this topic in paediatric age, especially because it gives clinicians the message to plan a targeted level of sedation for each patient and to regularly review it.

In 2014, an expert panel of the Italian Society of Neonatal and Pediatric Anesthesia and Intensive Care (SARNePI) developed 37 evidence-based recommendations [2]. Two years later, the Pediatric Cardiac Intensive Care Society produced a consensus statement dedicated to pharmacotherapy in cardiac critical care and focused on sedation, analgesia, and muscle relaxant treatment [3]. In 2016, the European Society of Pediatric and Neonatal Intensive Care (ESPNIC) published a position statement to recommend accurate monitoring of pain and non-pain-related distress in neonates and children admitted to the intensive care unit (ICU) and adoption of validated tools to evaluate the level of sedation, iatrogenic withdrawal syndrome (WS), and delirium [4].

However, nowadays a standardized approach to analgesia and sedation in Italy does not exist and single centres follow internal protocols, with high heterogeneity across the country. Through the development of national recommendations, the variations in practice may be reduced, thus improving patient care.

The present document was produced through a consensus of national experts in intensive care. It covers many different perspectives, providing recommendations and research priorities.

These recommendations aim to assist clinicians and nurses in performing adequate analgesia and sedation to 0–18 aged critically ill children (excluding neonates). The document is intended both for paediatric and adult intensivists who care for children admitted to intensive care units.

## Main text

### Materials and methods

#### Selection of the panel members

In May 2018, the SARNePI appointed two co-chairs (coordinators of the SARNePI Pediatric Neurological Protection and Drugs study group) to carry out this project. The selection of panel members was conducted by the co-chairs, considering expertise in analgesia and sedation or in associated aspects. The panel was composed of 13 paediatric intensivists recruited from 12 paediatric intensive care units (PICUs), a paediatrician, a neuropsychiatrist, a psychologist, a neurologist, a pharmacologist, an anaesthesiologist, two critical care nurses, and a methodologist. Out of a group of 22 panellists, 16 (13 paediatric intensivists, 2 critical care nurses, a paediatrician) participated in the development of consensus on recommendations.

All panel members declared any potential conflicts. All of them had no conflict of interest to declare.

#### Question development

In 2018, following the National regulation for Guideline production, the panel convened a meeting to discuss the project and to identify the main clinical topics. Seven areas of interest (topics) were initially considered: analgesia and sedation, difficult analgesia and sedation, neuromuscular blocking, sleep, delirium, withdrawal syndrome, palliative sedation. During the second meeting, one additional topic was added: analgesia and sedation in children with developmental delay. To consider the family’s perspective, a section with the caregivers’ opinion was included, assessed through a questionnaire administered in 4 PICUs. For each topic, the working group identified the deemed clinically relevant research questions, according to the PICO format, which describes the population (P), intervention (I), control (C), and outcomes (O). After finalization of the research questions, a non-weighted poll was performed by the panel to decide the priority questions.

#### Search strategy and evidence summation

For each PICO, a search strategy of literature was formulated. Search adopted a combination of controlled vocabulary (e.g. “pediatrics,” “pediatric critical care”, “intensive care unit”) and keywords (e.g. “pain: acute, measurement, assessment, management”, “sedation”, “deep sedation”, “conscious sedation”, “iatrogenic withdrawal syndrome”, “abstinence syndrome”, “substance withdrawal syndrome”, “delirium”) selected according to the research question. Keywords were combined with “AND” pediatric critical care “OR” Intensive Care Unit.

PUBMED, SCOPUS, EMBASE, COCHRANE, CINHAIL databases were considered.

All studies were selected using the filter of age (birth to 18 years) and language (English). Studies including only neonates were excluded.

The search was applied to the following period: January 1, 2008–January 1, 2020. For some topics, papers older than 2008 were included due to the paucity of literature.

Panellists selected literature considering systemic reviews or meta-analysis, randomized controlled trials (RCT), observational studies, case series, case reports, narrative reviews were appropriated. Editorials and letters to the Editor were excluded. Studies published after the strategy search date were not included.

The panel members, working blinded in couples, performed the selection of the literature based on title and abstract, including all the studies selected by one or both the reviewers.

**Fig. 1:**
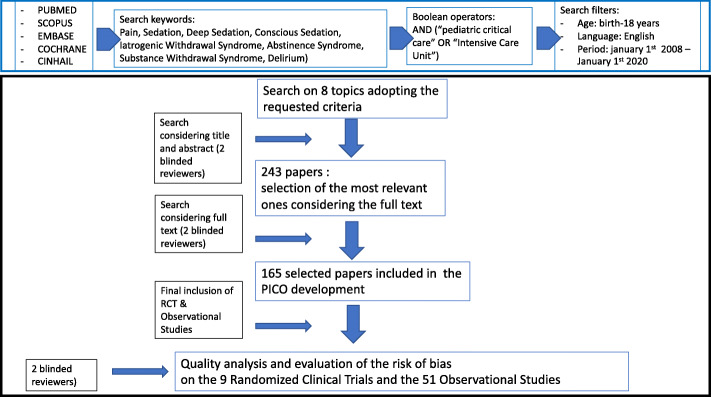
Selection of literature

After the first selection, 243 papers were evaluated with a full-text analysis by the panel members working blinded in couples. The agreement between the pairs was achieved through an electronic-based discussion. Finally, 165 relevant studies were selected for the PICO development (Fig. 1).

Nine RCT and 51 non-randomized observational prospective studies were considered for the data extraction.

#### Data extraction

For each selected study, a form was completed with the following details: general study information (first author, title, journal, year PMID), study design characteristics (design, setting, period, country), participant characteristics (age, diagnosis), interventions (intervention/method control group/comparison group), outcome characteristics (research question, primary and secondary end points), main results, measurements, data analysis, strengths, and limitations (see Supplementary Material 1a,1b,1c,1d,1e,1f,1g,1h).

#### Quality appraisal

Each selected study was assessed to evaluate the risk of bias by panel members, working blinded, in couples. The Cochrane risk of bias tool was adopted for randomized trials [5] considering selection bias, reporting bias, other bias, performance bias, detection bias, attrition bias.

The Newcastle-Ottawa Scale [6] was adopted for non-randomized prospective observational studies, considering selection bias, comparability bias, and outcome bias.

The final agreement between the pairs was achieved through an electronic-based discussion without the need for a third reviewer (see Supplementary Material 2).

Due to the paucity of the literature, it was not possible to summarize the quality of evidence for combined outcome.

#### Synthesis of the results

For each topic, a table with the characteristics of the included (general study information, study design characteristics, participant characteristics, interventions, outcome characteristics, main results, measurements, data analysis, strengths, and limitations) was prepared.

For each PICO, a narrative synthesis of the literature was reported, followed by the recommendation (see below).

#### Formulation of recommendations

PICO development was discussed during the third meeting. Seventeen recommendations were formulated, 16 on the 8 topics and one on a special issue (communication) added to the text and related to caregivers’ opinion. For each recommendation, were reported (see Supplementary Material 3): the level of evidence, the strength of recommendation, the benefits, the harms and risks, the benefit-harm balance, the intentional vagueness (if applicable), the value judgement, the exclusions (if applicable), the difference of opinion between the panel members, the knowledge gaps and research opportunities.

To designate the levels of recommendations, the panel followed the American Academy of Pediatrics policy statement “Classifying Recommendations for Clinical Practice” [7] with the application of the Evidence-to-Decision (EtD) framework according to the Grading of Recommendations Assessment, Development and Evaluation (GRADE) approach.

Recommendations were developed by the two chairs considering published literature, following an evidence-based approach to provide evidence-based recommendations, considering risks and benefits and applicability. Recommendations should have been discussed during the fourth meeting but, due to the corona virus disease-2019 lockdown, the members used telematic communication to reach a final agreement using a Delphi-modified technique to obtain the consensus on the recommendations and on the strength of the recommendations. For each recommendation, 100% of consensus was reached. The strength of recommendations was finally classified as strong, moderate or weak. Strong recommendations started with “We recommend”, moderate or weak recommendations started with “We suggest”. Concerning the strength of recommendation, 75% of the consensus was considered.

#### Quality assessment of recommendations

These recommendations were developed considering the AGREE II (Appraisal Guideline REsearch Evaluation) Reporting Checklist [8].

The draft version of this document underwent peer review and approval by representatives of the following scientific societies: SARNePI, Italian Society of Analgesia Anesthesia and Intensive Care (SIAARTI), Medical and Nursing Academy of Paediatric Emergency and Intensive Care (AMIETIP), and National Association of Critical Care Nurse (ANIARTI). Comments were reviewed by the co-chairs and incorporated into the recommendations as appropriate.

We planned to revise the recommendations every 5 years with the possibility to update them earlier if relevant literature on the topics will be published.

#### Dissemination and implementation

Dissemination of these recommendations will occur by publication in the websites of relevant scientific societies, national and international journals, presentations at national and international conferences, education sessions, and meetings with staff at the individual institution level to assess the need for local adaptation.

To assess the impact of the recommendations on the care of patients, we design to launch a national survey one year after the publication of the document, particularly analysing the following indicators: incidence of withdrawal syndrome, incidence of delirium, rate of monitoring of the level of sedation, withdrawal syndrome, delirium, adopting respectively the Comfort Behaviour Scale (CBS), the Withdrawal Assessment Tool-1, (WAT-1), and the Cornell Assessment Pediatric Delirium (CAPD).

### Summary of recommendations

1. As a first-line strategy, we suggest optimizing analgesia using opiates and adopting alpha agonists as sedative agents, considering benzodiazepines a second-line.
2. We suggest adopting protocols of analgesia and sedation to administer the minimal effective dose of analgesics and sedatives to reduce tolerance and the incidence of difficult analgesia/sedation. Furthermore, the daily interruption of sedation should be considered with caution.
3. We recommend regular monitoring with validated tools the level of analgosedation of paediatric patients admitted to ICU.
4. In difficult analgesia/sedation, we suggest using ketamine, due to its good safety profile.
5. We suggest using neuromuscular-blocking agents in patients with severe respiratory insufficiency and persistent patient-ventilator asynchrony despite actions taken to limit the rate of asynchrony.
6. We suggest monitoring the level of sedation with continuous processed EEG in patients treated with neuromuscular-blocking agents, considering the limitation and availability of the device.
7. We recommend adopting in all paediatric patients admitted to ICU strategies to prevent sleep alterations, particularly non-pharmacologic ones (relaxing techniques, parental involvement, control of environmental factors).
8. We recommend working on modifiable risk factors, particularly reducing the use of benzodiazepines.
9. We suggest basing the treatment of paediatric delirium on maximizing preventive bundles. Antipsychotic drugs may be used with careful consideration of contraindications.
10. We recommend regular monitoring delirium in critically ill children every day of the ICU stay, using validated tools.
11. We recommend working on modifiable risk factors of WS, particularly avoiding weaning higher than a daily reduction of 20% with respect to the initial dose.
12. We recommend treating WS with additional boluses of the drug considered to be responsible for the symptoms and modifying the weaning plan.
13. We recommend regular monitoring withdrawal symptoms in critically ill children treated with analgesics and/or sedatives longer than 72 h, adopting validated tools.
14. We recommend performing paediatric palliative sedation (PPS) early defining an interdisciplinary plan agreed with parents.
15. We suggest adopting a personalized strategy to achieve PPS in children, to ensure the maximal efficacy using doses tailored to the patient.
16. In children with developmental delay, we suggest adopting validated tools to monitor the level of sedation, the presence of delirium, and WS in ICU, considering their limitations and involving the caregivers.
17. We recommend explaining to parents the meaning of analgesia and sedation and off-label drugs. If analgesia and sedation lasted more than 48 h, we recommend informing parents about the risk of WS and delirium development.

**I. Analgesia and sedation**

Children admitted to the intensive care unit are exposed to pain due to clinical situations and procedures. An adequate level of sedation may be reached only if pain is absent. Acute pain needs to be immediately identified by physicians and nurses [4]; otherwise, it may become chronic. Chronic pain is difficult to treat and requires a specific approach [9].

Clinicians need to plan an “ideal” goal of sedation for each patient. Generally, a state of a quiet, sleepy child requiring stimulus to being awake without distress and excessive movements is considered an adequate level of sedation during mechanical ventilation.

Adequate analgesia and sedation depend on pharmacologic and non-pharmacologic treatment, but also environmental factors [10].

Over-sedation is correlated with increased length of ventilation and ICU stay, with increased incidence of WS and delirium. Under-sedation is correlated with patient’s discomfort and adverse events related to the risk of devices removal [11].

*Which is the best pharmacologic treatment to ensure an adequate analgesia and sedation in children in ICU?*

Opiates are the most common analgesic drugs adopted in the paediatric intensive care unit (PICU), especially morphine and synthetic opiates (fentanyl, remifentanil, sufentanil). Remifentanil seems to promote a more rapid weaning from ventilation compared to fentanyl, but, due to its ultra-brief half-life, the risk of developing tolerance and hyperalgesia increases, particularly with high doses [12]. Some authors consider remifentanil the ideal opiate in ventilated infants, due to a low risk of accumulation. In a single-centre, double-blind RCT, the median extubation time was significantly shorter in the remifentanil group but results are strongly conditioned by the low sample size [13].

Genetic polymorphism promotes the individual variability of drug response and the possibility to develop chronic pain. Facilitating and protective genotypes were identified, some of them correlated with the efficacy of analgesic drugs, others to the development of toxicity. A recent review reported 10 genotypes involved in individual response to opiates [14]. In particular, the genotype ABCB1 seems to be involved. Children with allele AA are described to require a lower dose of fentanyl compared to AG or GG alleles [15]. Recent studies on morphine and midazolam in ventilated children reported a possible role of UGT2BT polymorphism in midazolam metabolism [16, 17]. Therefore, genotype identification may be relevant to predict dosing requirements and treatment efficacy.

For perioperative pain management, in 2018 a clinical guidance (ESPA Pain management Ladder) was published by the European Society for Paediatric Anesthesiology [18].

Considering sedatives, in PICU the most used drugs are benzodiazepines, particularly midazolam. Italian data were reported by Tabacco et al. [19]

However, concerns about benzodiazepines have recently emerged. Drugs acting on γ- aminobutyric acid (GABA) receptor might promote a neurotoxic effect especially in patients younger than 3 years [20, 21]. Moreover, a direct and dose-dependent association of benzodiazepine with the development of delirium in critically ill children has been reported [22, 23].

At present, barbiturates were used in status epilepticus, in procedural sedation [21], and in paediatric brain trauma with refractory intracranial hypertension [24].

In the last years, the use of alpha-agonists as adjuvants increased, also in Italy [25]. In 2015, the Italian Medicines Agency (AIFA) admitted the use of dexmedetomidine in critically ill children refractory to conventional treatment [26]. Wolf et al, in a double-blind RCT, considered efficacy and safety reporting a non-inferiority of clonidine versus midazolam [27]. Recently, interest turned to dexmedetomidine, a drug with more specific stimulation of alpha 2 receptors. A review published in 2016 reported a sparing effect of dexmedetomidine compared to opiates and benzodiazepine, with a good safety profile [28]. In the same year dexmedetomidine was evaluated in a large study on paediatric ventilated patients, analysing the use of this drug as first-line therapy, as second-line therapy, and as a before-extubation strategy. In the group of patients treated with dexmedetomidine as first-line therapy a good level of sedation (measured with a validated scale) was reached. The use of dexmedetomidine before extubation was effective in reducing the length of ventilation [29]. In children submitted to cardiac surgical procedure, this result was confirmed [30]. A retrospective large monocentric study reported a good efficacy of dexmedetomidine as a single sedative agent in non-invasive ventilated paediatric patients [31].

**Recommendation 1:**

As a first-line strategy, we suggest optimizing analgesia using opiates and adopting alpha agonists as sedative agents, considering benzodiazepines a second-line.

**Strength of recommendation:** Moderate

*Are protocols of analgosedation useful in children admitted to ICU?*

Data on adults reported the efficacy of protocols of analgesia and sedation in reducing complications of ICU stay. In the paediatric population their efficacy is less strong and only a few studies have proven a positive impact of nurse-driven protocols. In a multicentre cluster-randomized trial, the use of a sedation protocol did not reduce the duration of mechanical ventilation. Nevertheless, patients in the intervention group were exposed to fewer days of opioid exposure and sedative classes [32]. Some authors compared phase before and after protocol implementation, showing a reduction in analgesic and sedative doses and in tolerance development of tolerance.

Particularly, after protocol implementation were reported: a reduction in the duration of treatment with opiates and benzodiazepine [33], a reduction in the incidence of WS [3, 34–36], a reduction of daily doses of benzodiazepine and a reduction in the duration of mechanical ventilation in children older than 12 months [37], a reduction of cumulative doses of benzodiazepine and WS in surgical patients [38]. Nevertheless, the only implementation of a protocol itself does not improve the quality of care for a prolonged time, because reinforce strategies need to be maintained [33, 39].

In adults admitted to ICU, daily interruption of sedation was reported to decrease the duration of mechanical ventilation and of ICU stay. Cumulative doses of benzodiazepines also decreased. In the paediatric population RCT are scarce and their results are questionable. The first RCT showed a reduction of the length of mechanical ventilation, PICU stay and cumulative dose of midazolam in patients treated with interrupted sedative infusion versus continuous infusion [40]. Conversely, a later RCT reported that daily interruption of sedation does not obtain these results but promotes more frequent periods of under-sedation [41]. In a more recent RCT, daily sedation interruption was evaluated in addition to protocolized sedation, finding that daily sedation interruption did not reduce the duration of mechanical ventilation, ICU stays, or amounts of sedatives, and was associated with increased mortality. For this reason, the authors don’t recommend this strategy of treatment [42].

Nowadays, protocols based on drug rotation of analgesic and/or sedatives don’t find the support of the literature.

**Recommendation 2:**

We suggest adopting protocols of analgesia and sedation to administer the minimal effective dose of analgesics and sedatives to reduce tolerance and the incidence of difficult analgesia/sedation. Furthermore, the daily interruption of sedation should be considered with caution.

**Strength of recommendation:** Moderate

*Which is the adequate monitoring of analgesia and sedation in paediatric patients admitted to ICU?*

It is challenging to distinguish between pain, distress, WS, and delirium in critically ill children because symptoms—depending on patient’s age—are often not specific and may overlap. For this reason, it is of priority importance to include in every protocol of analgesia and sedation regular monitoring of patients with validated scales to evaluate the level of analgesia, sedation, the presence of WS and delirium [4, 21]. Therefore, an educational plan and sensibilization of health care professionals, particularly nurses, due to their central role in using monitoring tools, need to be implemented in the ICUs.

Validated scales adopted in critically ill children to evaluate the level of analgosedation are:

- the CBS, a scoring system based on observational and behavioural variables, to evaluate distress (including pain) in critically ill children [43, 44]. The CBS indicates a range of adequate sedation and scores of over- and under-sedation. It may be used also in post-surgical ventilated children, including cardiac patients. The ESPNIC strongly recommends adopting this tool to monitor every 4–8 h the level of sedation in critically ill children admitted to ICU.
- the State Behavioural Scale, a 6-level scoring system to evaluate patient’s level of sedation with respect to his/her planned goal [45].
- the Richmond Agitation-Sedation Scale, a 10-level scoring system to evaluate patient’s level of sedation with respect to his/her planned goal [46].
- the FLACC (Face, Legs, Activity, Cry, Consolability), the WONG BAKER and the Numeric Rating (NRS) scales used (depending on patient’s ages) to specifically evaluate and monitor pain [47–49].

Regular use of these scales may increase workload but promotes the improvement of quality of care.

**Recommendation 3:**

We recommend regularly monitoring with validated tools the level of analgosedation of paediatric patients admitted to ICU.

**Strength of recommendation:** Strong

**II. Difficult analgosedation**

At present, no unique criteria exist to define difficult analgosedation. Recently, Lebet et al. conducted a survey between experts, to design a model to predict difficult analgosedation, dividing associated variables in three groups [50].

**Variables related to sedation:** need to use more than three sedative drugs, presence of inadequate sedation lasting more than 2 h, need to increase sedative doses higher than 90° centiles considering usual starting dose, need to administer to the patient intermittent doses of neuromuscular-blocking agents to adapt him/her to ventilator.

**Variables related to adverse events:** suspected delirium, unplanned extubation, unplanned removal of invasive devices, paradoxical response to sedation.

**Demographic/diagnostic variables:** 21 trisomy, previous sedations, non-communicating patients, Body Mass Index higher than 90° centile, cancer disease, moderate disability, bronchiolitis.

Some of these variables were reported by Mencia et al, adding the presence of prolonged mechanical ventilation [51].

*Which analgosedative drugs are useful in difficult analgosedation?*

In difficult analgosedation it is indicated to use third-line drug strategy, including molecules acting on GABA or N-methyl-d-aspartate (NMDA) receptor, with possible risks of neurotoxicity.

Ketamine continuous infusion may have a role as an adjuvant to children difficult to sedate. Nowadays, prospective studies on ketamine in PICU are lacking [52]. The review of Golding et al, including only case report series, reported the achievement of satisfactory sedation and analgesia by using ketamine, with an improvement of pulmonary compliance and oxygenation, and minimal adverse effects (nystagmus, flushing, one report of hypertension) [53].

Ketamine showed a good safety profile particularly in patients with bronchospasm, thanks to bronchodilator effects of this drug [54].A retrospective study published in 2017 reported that ketamine may reduce the development of opioid tolerance [55]. In a second retrospective study published in 2019, ketamine, administered in continuous infusion, did not impact mortality and haemodynamic stability of critically ill children [56].

Propofol, according to Italian Medical Agency (AIFA) recommendations, should not be used in paediatric ages in continuous infusion, particularly due to the risk of development propofol infusion syndrome. Nevertheless, a dose less than 4 mg/kg/h has been reported safe if the infusion lasts no more than 48 h [57].

Sevoflurane has been used in PICU as an adjuvant to morphine infusion, with the AnaConDa system, obtaining weaning from other sedatives in most patients [50]. A recent prospective study confirmed its efficacy in weaning from sedation and ventilation in critically ill children ready to extubation but very agitated and affected by WS [58]. Nowadays, the most effective dose and tolerated duration of treatment are not clear.

Few studies exist on the efficacy of levomepromazine as a sedative agent in paediatric population admitted to ICU. At present, it could be used in agitation states refractory to other treatments [59, 60].

**Recommendation 4:**

In difficult analgesia/sedation we suggest using ketamine, due to its good safety profile.

**Strength of recommendation:** Weak

**III**. **Neuromuscular-blocking agents**

*Which is the indication to the use of neuromuscular-blocking agents in children admitted to ICU?*

Neuromuscular-blocking agents (NMBA) in critically ill children are limited to specific indications: to guarantee patient’s immobility, to adapt patients to mechanical ventilation, to obtain a reduction of oxygen demand. Moreover, NMBA are used during the procedure of endotracheal intubation. NMBA are wieldy, show predictable pharmacokinetics also in children, and did not report severe adverse effects. Moreover, sugammadex, an antagonist of rocuronium bromide with a good safety profile in infants and children, gives clinicians the possibility to obtain an immediate reversal effect.

The Pediatric Cardiac Intensive Care Society does not report specific indications of using NMBA, but underlined that hyponatremia, hypokalemia, and hypocalcemia may increase their effect whereas hypermagnesemia may decrease it [3].

The Pediatric Acute Lung Injury Consensus Conference recommends the use of NMBA in patients with pediatric acute respiratory distress syndrome and adequately sedated, to adapt patients to mechanical ventilation, to decrease doses of sedative drugs, and to obtain a reduction of oxygen demand and respiratory effort [61].

A prospective study on children ventilated more than 24 h and treated with NMBA in continuous infusion reported a reduction of the thickness of diaphragmatic muscle, but the clinical impact of these data needs to be confirmed [62]. A secondary analysis of an RCT on therapeutic hypothermia showed the use of NMBA does not impact mortality and morbidity of critically ill children [63]. A recent case-control study reported an increase in the incidence of infections in patients with acute kidney insufficiency treated NMBA [64].

**Recommendation 5:**

We suggest using neuromuscular-blocking agents in patients with severe respiratory insufficiency and persistent patient-ventilator asynchrony despite actions taken to limit the rate of asynchrony.

**Strength of recommendation:** Weak

*Which is the adequate monitoring of analgosedation in paediatric patients admitted to icu and treated with neuromuscular-blocking agents?*

In patients treated with NMBA, monitoring the level of sedation is a priority and a challenge. Patients are immobilized, thus observational scales cannot be used. In paediatric patients, the interruption of NMBA infusion with the aim to monitor patients with validated observational scales may promote adverse events [61].

At present, continuous processed electroencephalogram (cpEEG) is the most used and studied tool in adults, but few studies exist in paediatric patients. An RCT performed in the operating room evaluated the effect of mivacurium on Bispectral Index and Cerebral State Index reporting no modification of the scores after NMBA infusion and suggesting the possibility to correctly monitor the level of sedation adopting cpEEG [65]. Even though that cpEEG is not validated to evaluate the level of sedation in paediatric patients (particularly infants) treated with NMBA and its availability is still limited, we suggest adopting this tool in critically ill patients if observational scales are not applicable. However, the correct use of cpEEG requires specific skills in analysing the trace and in understanding the score.

**Recommendation 6:**

We suggest monitoring the level of sedation with continuous processed EEG in patients treated with neuromuscular-blocking agents, considering the limitation and the availability of the device.

**Strength of recommendation:** Weak

**IV. Sleep**

Characteristics of sleep change with age and neurological development, both in duration than in frequency of a phase with respect to the others [66].

ICU admission exposes patients to environmental factors, procedures and drugs, causing a modification of physiologic phases of sleep. Most alterations of sleep architecture were reported in ventilated children [67].

In burned paediatric patients admitted to ICU, sleep modification was frequently reported [68, 69]. Pain, fear, anxiety, often present in burned patients, promote sleep negative effects, especially in younger children. Most analgesic and sedative drugs (opiates, benzodiazepine, ketamine, propofol) reduce slow-wave sleep phases of the non-rapid eye movement period and rapid eye movement (REM) period, whereas stage 1 increases, with frequent arousals [66, 69, 70].

Sleep modifications develop in ICU and persist after patient’s discharge [71]. Alterations of sleep architecture or sleep duration impact many physiologic mechanisms promoting delirium or neurocognitive/neuro-psychiatric long-term sequelae, immunodepression, metabolism and respiratory insufficiency, thus preventing patient’s recovery and patient’s weaning from mechanical ventilation.

Despite the evidence of a negative impact on patient’s outcome caused by sleep modifications, only recent studies covered this research area in paediatric population admitted to ICU, also due to the presence of many confounding factors in this setting. Moreover, different tools to evaluate the quality of sleep exist: polysomnography (PSG), actigraphy, cpEEG, and methods based on the observation of patient’s behaviour.

PSG is the gold standard. However, this tool is not diffused in ICU and requires high competence to be used. Studies performed with PSG reported that structure of sleep is frequently modified in ICU: (1) time to achieve the first phase of sleep is longer (sleep latency), (2) duration of sleep (total sleep time) does not change but covers mostly the daytime hours, (3) duration of deepest phases (stage 3, slow–wave sleep), and REM phase, decreases; (4) duration of less deep phases (stage 1) increases; (5) arousals increases [72]. Moreover, in critically ill children EEG waves may be modified by the “brain injury” due to the clinical status and due to administered drugs. Finally, NMBA infusion makes PSG impossible to record patient’s muscular activity, required to analyse the electro-oculogram trace [67].

Actigraphy is a less complex tool with respect to PSG. However, it has not been validated in critically ill patients. It may be influenced by every movement (i.e. nursing procedure). Finally, actigraphy does not evaluate the quality of sleep.

cpEEG shows a good correlation with PSG, except for the possibility to identify alert states (wakefulness), periods after sleep onset (wakefulness after sleep onset), and REM phases [73].

Methods based on the observation of patient’s behaviour often do not correlate with PSG [67, 74].

*Which is the best strategy to optimize sleep in paediatric patients admitted to ICU?*

Pharmacologic treatment (melatonine and dexmedetomidine) to prevent and treat sleep alterations in ICU is not supported by evidence-based proof, in paediatric age.

In adults, some studies reported a positive effect of non-pharmacologic strategies, such as environmental modifications (particularly light control), on the suprachiasmatic nucleus and circadian rhythm [75–77].

In neonates, non-pharmacologic treatment, like the use of nests, hammocks, gentle touch, handling, protocols based on noise reduction and light control, parental involvement in patient’s care, showed an evidence-based efficacy on quality of sleep [78]. It is reasonable that also in paediatric patients these strategies might produce the same results.

Finally, some positive effects on duration and quality of sleep were reported using sequentially haloperidol and zolpidem on paediatric burned patients [79].

**Recommendation 7:**

We recommend adopting in all paediatric patients admitted to ICU strategies to prevent sleep alterations, particularly non-pharmacologic ones (relaxing techniques, parental involvement, control of environmental factors).

**Strength of recommendation:** Strong

**V. Delirium**

Delirium is an acute cerebral condition complicating outcome in critically ill children. According to *Diagnostic and Statistical Manual of Mental Disorders Fifth Edition* (DSM-5) by the American Psychiatric Association, delirium presents 5 key features: a disturb of consciousness and “awareness”, acute onset (hours to days) with a fluctuating course during the day, presence of other cognitive deficit (memory, language, visive-spatial, perceptive), criteria 1 and 3 are not dependent on pre-existing neurocognitive deficits or on a severe awareness deficit (like coma), evidence (history, physical examination, laboratory tests) that delirium is a consequence of a clinical condition, a substance intoxication, weaning from drugs, toxin or multifactorial factors [21, 80–82].

The physiopathology of delirium is complex. Three mechanisms seem to be particularly involved: neuroinflammation, modification of neurological mediators due to drugs administration, oxidative stress due to a clinical condition. The final result is a modification of neurological transmission with integration/processing of sensory inputs and motorial response.

In critically ill children three factors contribute to delirium development: clinical condition, pharmacological treatments, environmental factors [83, 84].

Three subtypes of paediatric delirium exist: hyperactive delirium (characterized by agitation, restlessness, hypervigilance, combative behaviour), hypoactive delirium (characterized by lethargy, deficit of attention, decreased response to stimulus), mixed delirium (characterized by hyperactive and hypoactive aspects). Hypoactive delirium is the most frequent subtype. It is correlated with a worse outcome. In a longitudinal study on paediatric delirium, hypoactive and mixed delirium were the most frequent subtypes (46.4% and 45.2% respectively) whereas hyperactive one was present in only 8.4% of patients. Children with delirium often show a modification of psychomotor activity, with delay in response to a stimulus or continuous agitation; moreover, emotional lability, inconsolable status, excessive quietude are present.

Frequently, the onset of paediatric delirium is during the first three days of ICU admission [82, 85–87]. Recent studies reported delirium in more than 20% of ICU admission. In a multicentric paper on 25 PICUs, the general prevalence of delirium was 25%, 53% in ventilated children. The highest prevalence was reported in critically ill children affected by inflammatory/infective diseases [88].

Finally, it must be considered that the presence of delirium increases costs of PICU admission, independently from duration of PICU admission, patient’s severity and age [89].

The presence of delirium promotes complications in paediatric critically ill patients. A prospective study showed a strong and independent association between paediatric delirium and mortality [85]. A previous study reported that paediatric delirium increases duration of ventilation and length of ICU stay [90]. Nowadays, no evidence exists on the presence of long-term cognitive disorders in children with a diagnosis of delirium during ICU admission. However, literature is scarce and the impact on caregivers has not been studied [91].

*Is it important to work on risk factors for paediatric delirium in ICU?*

Non-modifiable risk factors for the development of paediatric delirium are pre-scholar age, mechanical ventilation, cognitive deficit, congenital cardiopathy, hepatic insufficiency, the severity on admission, treatment with vasopressors or with antiepileptic drugs, and ICU stay longer than 5 days [85–87, 89, 92–94]. At present, other potential factors (burns, transfusions) need to be confirmed.

**Fig. 2:**
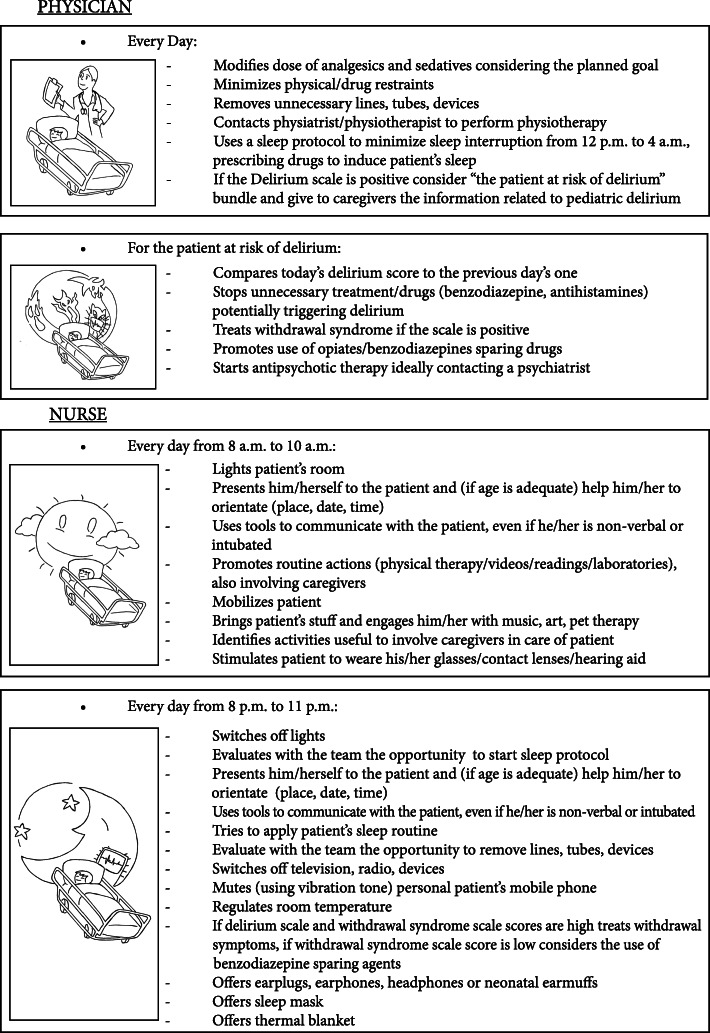
Bundle of paediatric delirium

Modifiable factors for the development of paediatric delirium are treatment with benzodiazepines (it increases from 2.5 up to 5 times the risk of developing delirium, with dose-dependent effect), use of restraint and patient’s immobilization, presence of noise, presence of light, modification of sleep–awake rhythm, absence of parents during ICU stay, treatment with anticholinergic drugs [22, 23, 82, 85, 87]. For this reason, preventive bundles for delirium are proposed with to reduce its incidence in critically ill children (Figure 2). However, their implementation may be hindered by structural problems or lack of resources.

**Recommendation 8:**

We recommend working on modifiable risk factors, particularly reducing the use of benzodiazepines.

**Strength of recommendation:** Strong

*Which is the best strategy to use in paediatric delirium?*

The pivot of strategy is represented by the identification and treatment of risk factors. Moreover, modifiable factors need to be considered and minimized [43, 95].

Great attention should be paid to create a familiar and comfortable environment, with light and noise reduction. Strategies to prevent delirium are usually displayed in “bundles” [96, 97]. If a patient presents delirium despite the preventive strategies, bundles application should be maximized.

Pharmacologic treatment follows adult therapies, due to the paucity and low quality of literature in paediatric age. Antipsychotic drugs, off-label for indication and, sometimes, age, in children may be used in selected cases. An electrocardiogram needs to be checked before treatment, particularly QT interval. A multidisciplinary approach is suggested, involving a neurologist or neuropsychiatrist.

These drugs are contraindicated if other molecules prolonging QT are administered to the patient or if patient suffers of severe cardiopathy or heart block. Finally, electrolytes should be regularly assessed [98].

According to a recent study, haloperidol reported adverse events even if the dosage was correct and blood level was below the therapeutic range, due to the occupation of dopaminergic receptor D2 [99]. Olanzapine, risperidone e quetiapine has been used in paediatric delirium with good efficacy in low-quality studies (retrospective ones, case series) [100–102].

Promising but anecdotal studies suggest a role of dexmedetomidine in the treatment of paediatric delirium [103].

**Recommendation 9:**

We suggest basing the treatment of paediatric delirium on maximizing preventive bundles. Antipsychotic drugs may be used with careful consideration of contraindications.

**Strength of recommendation:** Moderate

*Which is the adequate monitoring of delirium in paediatric patients admitted to ICU?*

At present, only a third of PICUs adopt tools to monitor delirium [104], despite validated scales exist. In 2011, following the Confusion Assessment Method for Intensive Care Unit (CAM-ICU) for adults, the Pediatric Confusion Assessment Method for the Intensive Care Unit (pCAM-ICU) was developed to monitor delirium in children older than 5 years [105]. In 2014, the Cornell Assessment of Pediatric Delirium (CAPD) was validated, including one year later “anchor points” to make the diagnosis of delirium in younger age and in children with developmental delay [106, 107]. To be applicable in the same groups of patients, in 2016 the Preschool Confusion Assessment Method for the ICU (psCAM-ICU), was developed [108].

In 2018, the Sophia Observation Symptoms-Pediatric Delirium (SOS-PD) scale was derived from the Sophia Observation withdrawal Symptoms scale (SOS); to monitor with a single tool both withdrawal syndrome and, thanks to the inclusion of items related to the cognitive and behavioural status, delirium. This scale may be applied in children older than 3 months [109].

All tools for the diagnosis of delirium include signs and symptoms of WS. Therefore, despite literature reported the presence of WS and delirium in the paediatric population, the overlap of signs and symptoms makes it difficult to distinguish between the two entities [110].

ESPNIC recommends adopting CAPD to monitor paediatric delirium and to educate health professionals working in PICU to identify it [4].

**Recommendation 10:**

We recommend regular monitoring delirium in critically ill children every day of the ICU stay, using validated tools.

**Strength of recommendation:** Strong

**VI. Withdrawal syndrome**

Prolonged infusion of opiates and benzodiazepines, through pharmacodynamic mechanisms of receptor desensitization and up-regulation of excitatory intracellular pathways, promotes tolerance. Tolerance is the need to increase doses to obtain a therapeutic effect. It is associated with dependence, that is the need to administer a substance to avoid withdrawal symptoms. WS is the set of symptoms due to an abrupt stop or a too fast weaning from drugs inducing tolerance and/or dependence. It is characterized by central neurologic system, gastrointestinal, and autonomic nervous system signs.

The incidence of opiates and benzodiazepine WS varies between 22.6 and 64.6% [111, 112]. This high incidence was reported in a multicentre prospective study performed in Italian PICUs [113].

WS developed after prolonged dexmedetomidine infusion overlaps symptoms of WS after opiates and benzodiazepines treatment [114, 115].

Patients with WS present a higher duration of mechanical ventilation and a longer ICU and hospital stay. At present, no studies exist on long-term neurocognitive sequelae after the development of WS [116, 117].

*Is it important to work on risk factors for paediatric ws in ICU?*

The most known risk factors for paediatric WS are drugs duration of infusion and administered cumulative doses [117, 118]. However, some studies reported the development of WS after infusion lasted less than 5 days [110, 111].

WS due to dexmedetomidine is reported after 48 h of infusion and no data exist on how to wean from this drug [115].

Opiates cumulative doses (including supplemental boluses) equal to or higher than 166.7 mg/kg (morphine equivalent) and benzodiazepines cumulative doses equal or higher than 60 mg/kg (midazolam equivalent) are considered at risk for WS. Recent studies reported, particularly for midazolam, the highest administered doses (0.3–0.4 mg/kg/h) as predictive for the development of WS [111, 112, 117].

Moreover, some modifiable risk factors were identified: the choice of drug (morphine seems to show a lower probability of risk than other opiates), adoption of multiple sedative drugs, daily weaning higher than 20% of the initial dose, high nurse workload. At present, demographic (youngest age) and clinical (neurocognitive delay) variables are not considered always risk factors of WS [111–113, 119, 120].

**Recommendation 11:**

We recommend to working on modifiable risk factors of WS, particularly avoiding weaning higher than a daily reduction of 20% respect on the initial dose.

**Strength of recommendation:** Strong

*Which are the best strategies in paediatric WS?*

The development of WS needs to be considered during all the analgesic and sedative treatment to minimize risk factors and work on modifiable ones.

Considering prevention, some studies reported the efficacy of nurse-driven protocols in reducing cumulative doses, particularly of benzodiazepines, through increased use of validated monitoring tools [34, 35, 37]. According to some authors, the use of alpha agonists may reduce analgesic and sedative cumulative doses [121, 122].

As first-line strategy of treatment, the modulation of the weaning plan should be implemented, eventually using additional boluses of the drug considered responsible for the symptoms.

Molecules adopted in neonatal age to prevent or treat WS are not considered evidence-based strategies in paediatric age [114]. No evidence-based data exist to support the role of receptor antagonists, like naloxone and dextromethorphan. Methadone, a synthetic opioid, may be used in opioid weaning with the aim of stopping the administered intravenous opiate in 24 h. Excessive sedation is the most frequent adverse effect of this drug [123, 124]. At present, the role of alpha agonists in treating WS needs to be confirmed. However, both clonidine as dexmedetomidine might have a potential action in preventing and treating WS, due to an opioid-sparing action mechanism and suppression of catecholamine release [125, 126].

**Recommendation 12:**

We recommend treating withdrawal symptoms with additional boluses of the drug considered to be responsible for the symptoms and modifying the weaning plan.

**Strength of recommendation:** Strong

*Which is the adequate monitoring of ws in paediatric patients admitted to ICU?*

Diagnosis of WS and evaluation of the efficacy of its treatment are challenging in ICU, due to the overlapping of symptoms of inadequate analgesia or sedation, delirium, discomfort induced by environmental factors (noise, light…), or other pathological conditions. Moreover, opiates and benzodiazepines withdrawal symptoms overlap [110].

ESPNIC identified two monitoring tools for opiates and benzodiazepine WS, validated in critically ill children:

- WAT-1
- SOS

WAT-1 was the first developed tool, validated in paediatric age. WAT-1 and SOS showed similar sensibility and specificity, whereas modality of application, numbers of items, and patient’s stimulation are different [4, 127–129].

**Recommendation 13:**

We recommend regular monitoring withdrawal symptoms in critically ill children treated with analgesics and/or sedatives longer than 72 h, adopting validated tools.

**Strength of recommendation:** Strong

**VII. Paediatric palliative sedation**

PPS is defined the intentional administration of sedative drugs to alleviate one or more refractory symptoms, with the aim of reducing the child’s awareness at the end of his/her life [130]. A symptom is refractory when any possible treatment fails or if no method is available to alleviate it in a reasonable time a dying child may tolerate [131]. The most common refractory symptoms at the end of life in paediatric age are pain, dyspnoea, fatigue, delirium, seizures, and vomit [132].

Definition of PPS does not intentionally distinguish between continuous and intermittent sedation, between light and deep sedation, considering the “proportionality” the ethical pivotal rationale [133].

The aim of PPS isn’t to promote death during the terminal phase of life, but to alleviate pain and distress due to refractory physical symptoms if no other therapeutic option exists [134].

*Which are the organization strategies to perform an adequate PPS?*

To care and to plan the end of life are crucial, not only for the child but also for parents. Parents will keep in their mind last the period of his/her child’s life forever.

A clear and multidisciplinary plan of care must be established. It needs to be agreed with parents after informed consent, indicating to stop/not to start new supporting therapies with to permit the natural patient’s death. All the figures caring for the child need to be involved: paediatricians, intensivists, palliativists, neonatologists, oncologists, pneumologists, neurologists, neuropsychiatrists, psychologists, social workers [134–136].

At present, most incurable children die in ICU, despite the fact this setting is not appropriate for many reasons [137, 138].

In Italy, law 38/2010 and 219/2017 declares as human right the possibility, if refractory symptoms are present, to receive palliative sedation, making PPS is accepted from an ethical point of view [139, 140].

**Recommendation 14:**

We recommend performing PPS early defining an interdisciplinary plan agreed with parents.

**Strength of recommendation:** Strong

*Which is the therapeutic strategy to obtain an adequate PPS?*

At present, a unique therapeutic strategy to conduct PPS does not exist, but recommendations of experts and single-centre protocols are reported [141].

If the patient shows extreme agitation, anxiety, or dyspnea, the use of midazolam associated with morphine is considered in many publications the first-line strategy, adopting variable doses [142]. Morphine is used as a single drug in 25% of cases and associated with midazolam in 50% [143].

As second-line or third-line therapy, neuroleptics, barbiturates, propofol and recently dexmedetomidine were administered if midazolam was not effective or when delirium is the refractory symptom to treat [103]. Some experts in this setting recommend the use of haloperidol, cited in WHO 2008 list of necessary drugs in paediatric palliative care, to reduce midazolam [134, 144]. Single-centre experience reported as effective the use of propofol particularly in adolescents or if the first-line strategy was ineffective. Doses of propofol reported in the literature are in the range between 0.5 and 16 mg/Kg/h; duration of infusion has been described up to 30 days [130, 145–148]. Ketamine has been used in children to control severe pain, with the aim of decreasing opioids escalation or reducing neuropathic pain [149–151].

The pivotal is to obtain patient’s comfort, increasing doses of drugs and evaluating their efficacy. Analgesic and sedative doses in PPS are generally higher with respect to them reported in the summary of product characteristics. Vital sings monitoring during PPS is not recommended [131].

**Recommendation 15:**

We suggest adopting a personalized strategy to achieve PPS in children, to ensure the maximal efficacy using dosed tailored to the patient.

**Strength of recommendation:** Weak

**VIII. Analgesia and sedation in paediatric patients with neurodevelopmental delay**

In children with neurodevelopmental delay, pain response is often a maladaptive behaviour, with freezing, self-injury or modification of the usual daily skills [152].

*Which is the adequate monitoring of analgesia and sedation in paediatric patients with developmental delay admitted to ICU?*

Pain assessment is difficult due to the patient’s comorbidities and the level of neurodevelopmental delay, resulting in different expression and communication modalities [153]. Patients with cerebral palsy often show movement and posture abnormalities with seizures, muscular problems, sensorial, cognitive, communicative, or behavioural disorders. Therefore, the risk of underestimating the severity of pain or delaying the diagnosis in these patients is high, also due to the presence of many environmental disturbing factors. A recent secondary analysis of a randomized study reported, in children with developmental delay admitted to ICU, the use of a lower cumulative dose of analgesic and sedative drugs compared to the other patients. However, a higher incidence of WS was present. The authors underlined the risk of adopting inadequate monitoring tools to evaluate the discomfort in these children [154].

*Revised Face, Legs, Activity, Cry, Consolability* (r-FLACC) and *Non-Communicating Children’s Pain Checklist Postoperative Version (NCCPC-PV)* are validated scales for children with developmental delay [155, 156]. They are easily usable in the hospital also without the presence of caregivers. The Italian version is available [157, 158] but no validation in ICU exists. During the evaluation, the involvement of caregivers is necessary to better understand child’s behavioural and its modifications. More complex tools (*NCCPC-revised, Paediatric Pain Profile)* are diary-dossier of child’s history of pain with personalized scores to evaluate the severity of that patient’s pain during an event. These scales are not applicable in ICU. Finally, CBS was proposed to evaluate distress in children ages 0–3 years with Down syndrome, with good results [159].

To monitor delirium, authors of CAPD scale suggested the development of personalized “anchor points” for children with developmental delay, using caregivers’ collaboration during ICU admission [107].

Instrumental monitoring may be not accurate in these children, because seizure, cerebral disease, antiepileptic/neurological therapy may modify the data, as reported in a study performed during surgery in children with developmental delay, showing lower BIS scores than other children [160].

Patients with neurodevelopmental delay often need chronic drug treatment. Potential interactions between these therapies and analgesic and sedative drugs administered in ICU and their impact on patient’s safety should be considered [161]. At present, studies on this issue are lacking.

**Recommendation 16:**

In children with developmental delay, we suggest adopting validated tools to monitor the level of sedation, the presence of delirium and withdrawal syndrome in ICU, considering their limitations and involving the caregivers.

**Strength of recommendation:** Weak

*Which is the ideal communication to parents of critically ill children admitted to ICU in terms of analgesia and sedation?*

**The problem of the “off-label” analgesics and sedatives**

In the paediatric age, many drugs are administered considering the dose for adults. In these cases, no authorization from national or international drug agencies exists. For this reason, studies are highly required to gain pharmacologic data in neonates and children. Unfortunately, at present many drugs are off-label in paediatric age. Ethical, economic, biologic and patients’ physiologic factors make it difficult to promote studies related to this topic. Drug agencies developed skills and facilities to produce plans for paediatric research (PIPs). In 2017, the Committee report presented to Parliament and European Council, 10 years after UE Pediatric Regulation, showed an increased number of approved PIPs and authorizations for paediatric drugs, today “in label” [162]. Looking at European EudraCT database, clinical research increased, but a new approach to research, an alternative to clinical trials due to paucity of the sample (i.e. simulation models, extrapolation models…) needs to be developed.

In Italy, two laws regulate off-label drugs in the National Health System (NHS). The first one is the 648/1996 law, related to drugs used for not authorized indications if an alternative option is not available. The other law is 79/2014, related to drugs used for not authorized indications if an alternative option is available, when scientific data reported an economic advantage and appropriateness. Thanks to this regulation, after the official request from SARNePI, many analgesic and sedative drugs were authorized by AIFA: ketamine, morphine, fentanyl, alfentanil, remifentanil, propofol, midazolam, neuromuscular-blocking agents, local anaesthetics, antagonists like sugammadex, naloxone, and flumazenil and, more recently, dexmedetomidine [23, 163]. For each drug a specific authorized indications for the use were specified.

**Parents’ opinion**

Parents’ involvement is a priority for paediatric patients admitted to ICU. The approach to family, according to the model of family-centred care, is an efficient and always usable tool. Giving parents understandable information describing “what’s happening”, and “what the child will feel”, eventually using a cultural mediator, listening to parents’ perspective and point of view, respecting, if possible, parents’ desires and promoting parents’ participation in the patient’s care are necessary steps. Recent studies particularly underlined the importance of parents’ role in the prevention, evaluation and treatment of delirium [164] and WS [164, 165].

To evaluate parents’ opinions, in these recommendations we adopted an anonymous questionnaire with 6 questions related to analgesia and sedation. (see Supplementary Material 4) It was administered in 4 PICUs to 19 parents, during the discharge phase. Their child needed to be admitted longer than 48 h and analgesia and sedation needed to be administered during PICU stay.

Considering results (see below), satisfaction was good in 4/6 topic (questions 1, 2, 3, 5) and in 18/19 questionnaires.

**Fig. 3:**
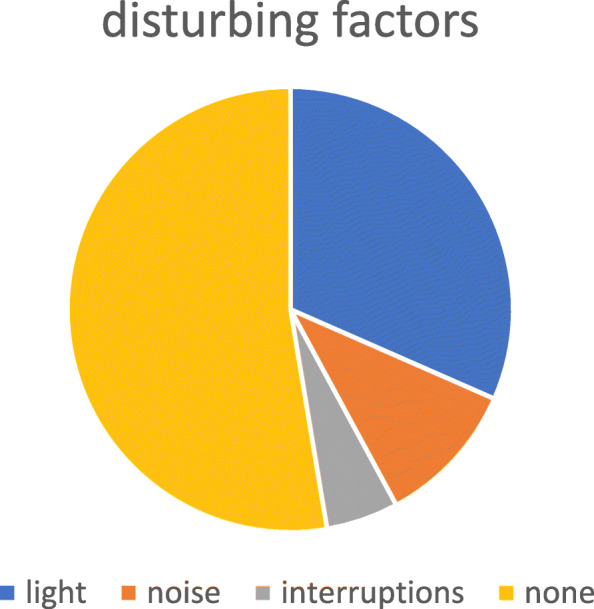
Parents’ opinions part A

**Fig. 4:**
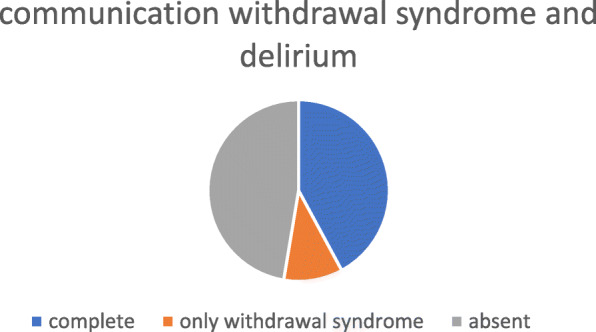
Parents’ opinions part B

Two areas of improvement were reported:

- Environment, considered disturbing in terms of lights, noise, interruptions (question 4) **(**Fig. 3)
- Communication related to WS and, particularly, delirium, considered inadequate (question 6) (Fig. 4)

**Recommendation 17:**

We recommend explaining to parents the meaning of analgesia and sedation and off-label drugs. If analgesia and sedation lasted more than 48 h, we recommend informing parents about the risk of withdrawal syndrome and delirium development.

**Strength of recommendation:** Strong

## Conclusions

In the paediatric age, the literature covering analgesia and sedation is poor and the quality of the studies is quite low. However, some relevant information has recently emerged. First, the indication to reduce the use of benzodiazepine as the first-choice sedative agent, preferring the alpha agonists, as an effective and safe option, associated with the opiates to cover the pain. Moreover, assessment is a key point to ensure the best care to patients, to maintain an adequate level of comfort and particularly to identify delirium and withdrawal syndrome. Four special aspects are developed in this document. The approach to patients with a development delay, which requires appropriate tools to be appropriately evaluated in terms of pain and sedation. The attention to the quality of patients’ sleep, not only to decrease the incidence of delirium. The significance of palliative sedation and the best strategy to achieve it in the PICU setting. Finally, the importance of complete communication to parents to explain the meaning of analgesia and sedation and its risks.

The list of research recommendations **(**Table 1) emphasizes how much it remains to be done. However, following shared recommendations remains the first step to offer the best analgesia strategy and sedation for critically ill children.

**Table 1:** Research recommendations

Recommendation 1	R1	*Studies on weaning from a prolonged infusion of dexmedetomidine*
Recommendation 2	R2	*Studies on the efficacy of drug rotation (analgesics or sedatives) protocols*
Recommendation 4	R4.1	*Studies on the prolonged infusion of ketamine*
	R4.2	*Studies on efficacy and safety of other drugs used in difficult analgesia and sedation (i.e., sevoflurane)*
Recommendation 5	R5	*Studies on length of treatment with neuromuscular blocking agents*
Recommendation 6	R6	*Studies on continuous processed EEG in infants*
Recommendation 7	R7.1	*Studies on preventing and treatment of sleep disorders in paediatric intensive care unit*
	R7.2	*Studies on monitoring the quality of sleep in paediatric intensive care unit with tools other than polysomnography*
Recommendation 8	R8.1	*Studies on the efficacy of alpha agonists in reducing the incidence of delirium in critically ill patients*
	R8.2	*Studies on the impact of reduction of modifiable factors in paediatric delirium*
Recommendation 9	R9	*Studies on pharmacologic prevention and treatment of paediatric delirium*
Recommendation 11	R11	*Studies on weaning after ketamine and alpha agonists infusions*
Recommendation 12	R12	*Studies on preventing withdrawal syndrome from adopting strategies of weaning or pharmacologic strategies*
Recommendation 15	R15	*Studies describing characteristics of paediatric palliative sedation in children in intensive care unit*
Recommendation 16	R16.1	*Studies on pharmacological interactions between patient’s chronic therapy and analgesic and sedative treatment administered in intensive care unit*
	R16.2	*Studies on the development of tools dedicated to children with developmental delay admitted to paediatric intensive care unit*

## Supplementary Information

**Additional file 1.** Synoptic Tables (files: Suppl Mat 1a, 1b, 1c, 1d, 1e, 1f, 1g, 1h).

**Additional file 2.** Risk of bias (file: Suppl Mat 2).

**Additional file 3.** Tables of Recommendations (file: Suppl Mat 3).

**Additional file 4.** Questionnaire for caregivers (filer: Suppl Mat 4).

**Additional file 5.** Drugs (file: Suppl mat 5).

**Additional file 6.** Drug interactions (file: Suppl mat 6).

## Data Availability

Not applicable.

## Abbreviations

AMIETIP: Medical and Nursing Academy of Paediatric Emergency and Intensive Care
ANIARTI: National Association of Critical Care Nurses
ESPNIC: European Society of Pediatric and Neonatal Intensive Care
CAPD: Cornell Assessment Pediatric Delirium
CBS: Comfort Behaviour Scale
FLACC: Face, Legs, Activity, Cry, Consolability
GABA: Aminobutyric acid
ICU: Intensive care unit
NMBA: Neuromuscular-blocking agents
PICO: population (P), intervention (I), control (C), and outcomes (O)
PICU: Paediatric intensive care unit
PPS: Paediatric palliative sedation
PSG: Polysomnography
REM: Rapid eye movement
RCT: Randomized controlled trials
SIAARTI: Italian Society of Analgesia Anesthesia and Intensive Care
SARNePI: Italian Society of Neonatal and Pediatric Anesthesia and Intensive Care
SOS Sophia: Observation withdrawal Symptoms scale
WAT-1: Withdrawal Assessment Tool-1
WS: Iatrogenic withdrawal syndrome
