# Supplementary material for: Recommendations for analgesia and sedation in critically ill children admitted to intensive care unit
Source: J Anesth Analg Crit Care. 2022 Feb 12;2:9. doi: 10.1186/s44158-022-00036-9 (PMC8853329; doi:10.1186/s44158-022-00036-9)
Supplement: Supplementary file 1 — Additional file 1. Synoptic Tables (files: Suppl Mat 1a, 1b, 1c, 1d, 1e, 1f, 1g, 1h). [file 44158_2022_36_MOESM1_ESM.zip › Additional file 1/JAACC Suppl Mat 1a Analgesia and Sedation.docx]

|  | First Author | Journal, Year,  PMID | Research Question | Design | Setting | Period (years)/Country | Patients/Age | Primary end-point | Secondary end-points |
| --- | --- | --- | --- | --- | --- | --- | --- | --- | --- |
| 1 | Welzing L | ICM 2012  22456770 | Efficacy and safety of remifentanil-based regimen for analgesia and sedation  in PICU | RCT  double-blind | Single-center PICU | November 2006-March 2010/Germany | 24 Pts/Neonates (≥36 EG)-young infants (<60 days)  Pts mechanically ventilated for at least 12-96 h | Extubation time following discontinuation of the opioid infusion | To compare efficacy and safety of remifentanil-based regimen to fentanyl-based regimen |
| 2 | Wolf A | Health Technology Assessment 2014  26099138 | Evaluation of intravenous clonidine as an alternative to intravenous midazolam as sedative agents in critically ill children | RCT  double-blind equivalence trial  SLEEPS study | Multi-centers  10 PICUs | November 2009-May 2012//UK | 129 Pts mechanically ventilated/30 days-15 years | Adequate sedation= COMFORT score 17-26 for ≥ 80% of the time  with a ±0.15 margin of equivalence | Percentage of time spent adequately sedated, increase in sedation/analgesia, recovery after sedation, side effects and safety data |
| 3 | Ista E | PCCM 2005  15636661 | Development of a scoring system to assess pain and distress: the COMFORT “behavioral” (COMFORT-B) scale | Prospective observational study | Single-center  PICU | March 2002-November 2002/The Netherlands | 78 Pts/0-18 years.  Exclusion Criteria: severe mental retardation, severe hypotonia or neuromuscular blockade | To assess whether physiologic variables (heart rate, mean arterial pressure) are really useful in the judgment of sedation with the COMFORT scale | To determine new cutoff points for a COMFORT scale restricted to behavioral items  COMFORT-B |
| 4 | Curley MAQ | PCCM 2006  16446601 | Development of a scoring system to assess sedation/agitation levels: State Behavioral Scale (SBS) | Prospective study | Single-center PICU | 2000-2004/USA | 91 Pts intubated and mechanically ventilated/6 weeks-6 year.  Exclusion Criteria: postoperative patients, neuromuscular blockade, in pain, unstable, at risk for withdrawal | To demonstrate preliminary construct validity and inter-rater reliability of the SBS |  |
| 5 | Grant MJC | PCCM 2016  27654816 | Dex use in PICU | Secondary analysis  of the RESTORE (RCT) database | Multi-centers  31 PICUs | June 2009-December 2013/USA | 2449 children with acute respiratory failure/2 weeks-17 years | To describe Dex use in PICU as primary agent (DEXp), as secondary agent (DEXs) or to facilitate endotracheal extubation (DEXe) |  |
| 6 | Kerson AG | J Intensive Care 2016  27800163 | Development of a scoring system to assess sedation/agitation levels: the Richmond Agitation-Sedation Scale (RASS) | Prospective study | Single-center PICU | USA | 50 Pts/2 months-21 years Exclusion Criteria: neuromuscular blockade, quadriplegia, impaired hearing and visual acuity | Validation of the Richmond Agitation-Sedation Scale (RASS) in critically ill children |  |
| 7 | Zuppa AF | Crit Care Med 2019  30920410 | Pharmacokinetics and pharmacogenomic of morphine in critically ill pediatric patients | Prospective observational study | Multi-centers  13 PICUs | USA | 66 Pts mechanically ventilated for acute respiratory failure/0.4-17.6 years. Inclusion Criteria: Pts receiving morphine continuous infusions, weight ≥7 Kg | To develop a PK-model to quantify the effects of critical illness or MV or genetic polymorphisms on morphine PK in PICU |  |
| 8 | Zuppa AF | Crit Care Med 2019  30672747 | Pharmacokinetics and pharmacogenomic of midazolam in critically ill pediatric patients | Prospective observational study | Multi-centers  13 PICUs | USA | 173 Pts mechanically ventilated for acute respiratory failure/0.32-18 years. Inclusion Criteria: Pts receiving midazolam continuous infusions, weight ≥7 Kg | To develop a PK-model to quantify the effects of critical illness or MV or genetic polymorphisms on midazolam PK in PICU |  |
| 9 | Best KM | J Pediatr 2018  30527750 | Analgesia and sedation need in critically ill children with neurocognitive impairment | Secondary analysis  of the RESTORE (RCT) database | Multi-centers  31 PICUs | June 2009-December 2013/USA | 412 Pts (17% of all RESTORE study PTS)/media age 6.2 years. Pts with pre-existing cognitive impairment had baseline PCPC ≥3 | To compare analgesia and sedation management between critically ill children with pre-existing cognitive impairment and critically ill neurotypical children | To compare indicators of therapeutic efficacy |

|  | Intervention/Method | Control Group/ Comparison group | Main Results | Measurements | Data Analysis | Strengths and limitations |
| --- | --- | --- | --- | --- | --- | --- |
| 1 | Remifentanil/Midazolam  11 Pts (8 Male) | Fentanyl/Midazolam  12 Pts (8 Male) | Extubation time was significantly shorter in the remifentanil group. Efficacy (scores in desired range) between two regimens was comparable. Safety (hemodynamic stability) profiles were similar.  No adverse events | Hartwig Sedation Score | Descriptive statistics. Comparison of variables by Mann-Whitney *u* test | Downgraded in quality assessment due to directness limitation: people of interest are older than those in the study |
| 2 | Clonidine/Morphine  65 Pts (43 Male) | Midazolam/Morphine  64 Pts (38 Male) | Equivalence was not demonstrated. Non-inferiority of clonidine to midazolam was established | COMFORT score | Comparison of variables by chi-squared test, Fishers exact test, Mann-Whitney *u* test. Kaplan-Meier survival analysis and log-rank test. Mix models for longitudinal data analysis.  Health economics analysis plan | Downgraded in quality assessment due to the low recruitment rate than the planned number |
| 3 | None  Nurses assessed Pts every 8-hr shift at set times |  | Physiologic variables do not correlate well with behavioral items of the COMFORT scale.  When COMFORT-B scores ≥11 and ≤ 22 adequate sedation cannot be based on COMFORT-B scores alone | COMFORT, Nurse Interpretation of Sedation Score (NISS) | Psychometric evaluation: interrater reliability, internal consistency, and concurrent validity were calculated |  |
| 4 | None  Nurses simultaneously assessed Pts |  | This study demonstrates preliminary construct validity and inter-rater reliability pf the SBS for use in Pts supported on mechanical ventilation | SBD, NRS | Psychometric evaluation: interrater reliability, construct validity. Subgroup analyses were made |  |
| 5 | Dex was prescribed according to protocol, was used in the peri-extubation period within a nurse-implemented goal-direct  sedation algorithm | Usual care: Dex use was unrestrained  *Most analyses focus on the usual care Pts* | Dex use increased during the period of the study. DEXp was used in low critically Pts and offered rapid achievement of targeted sedation. DEXs was used in high critically Pts and did not improve sedation profile. DEXe abbreviated ventilator weaning | FLACC scale, Wong-Baker FACES scale, INRS, SBS, WAT-1 | Multivariate statistics. Linear, logistic, multinomial logistic, cumulative logit regression models for continuous, binary, nominal, ordinal variables | Upgraded in quality assessment due to low imprecision |
| 6 | None  Nurses and researchers assessed the Pts |  | RASS is validated to assess levels of sedation and agitation in pediatric patients. It can be used to assess the responsiveness and to recognize hypoactive and hyperactive delirium | RASS, VAS, UMSS | Psychometric evaluation: interrater reliability, concurrent validity. Subgroup analyses were made |  |
| 7 | In Pts receiving morphine continuous infusion, blood samples were collected for drug quantification and genomic evaluation |  | Bodyweight and post-menstrual age are relevant predictors of PK parameters of morphine and its metabolites. Duration of MV ≥10 days reduces metabolite formation and elimination. The polymorphisms in UGT2B7 SNPs do not impact the metabolism of morphine | Plasma concentrations of morphine, morphine-3-glucuronide and morphine-6-glucuronide. Genotype identification | Serial blood sampling for drug quantification and single blood collection for genomic evaluation. Develop of PK and pharmacogenomic model |  |
| 8 | In Pts receiving midazolam continuous infusion, blood samples were collected for drug quantification and genomic evaluation |  | Bodyweight, age, hepatic and renal functions and UGT2B7 rs62298861 polymorphism are relevant predictors of midazolam PK variables. Midazolam clearance decrease with hepatic and renal dysfunction. Clearance increases in the minor allele | Plasma concentrations of midazolam, 1’ and 4’-hydroxymidazolam metabolites, 1’ and 4’ glucuronide metabolites. Genotype identification | Serial blood sampling for drug quantification and single blood collection for genomic evaluation. Develop of PK and pharmacogenomic model |  |
| 9 | Analgesic and sedative drugs prescribed according to protocol | Analgesic and sedative drugs prescribed according to unrestricted usual care/local practice norms | Adjusting for age and severity of illness, cumulative doses of opioids and BDZ were lower in Pts with cognitive impairment, they had more study days awake and calm, and more documented iatrogenic withdrawal symptoms | FLACC scale, Wong-Baker FACES scale, INRS, SBS, WAT-1 | Multivariate statistics |  |

Legend: BDZ: benzodiazepines; Dex: dexmedetomidine; FLACC: Face, Legs, Activity, Cry, Consolability pain scale; ICM: Intensive Care Medicine; INRS: Individualized Numeric Rating Scale; MV: Mechanical Ventilation; PCCM: Pediatric Critical Care Medicine; PCPC: Pediatric Cerebral Performance Category; PK: Pharmacokinetics; Pts: patients; RCT: Randomized Controlled Trial; UMSS: University of Michigan Sedation Scale; VAS: visual analog scale; WAT-1: Withdrawal Assessment Tool-version1.
