# Supplementary material for: Recommendations for analgesia and sedation in critically ill children admitted to intensive care unit
Source: J Anesth Analg Crit Care. 2022 Feb 12;2:9. doi: 10.1186/s44158-022-00036-9 (PMC8853329; doi:10.1186/s44158-022-00036-9)
Supplement: Supplementary file 1 — Additional file 1. Synoptic Tables (files: Suppl Mat 1a, 1b, 1c, 1d, 1e, 1f, 1g, 1h). [file 44158_2022_36_MOESM1_ESM.zip › Additional file 1/JAACC Suppl Mat 1b Difficult A S .docx]

|  | First Author | Journal, Year,  PMID | Research Question | Design | Setting | Period (years)/Country | Patients/Age | Primary end-point | Secondary end-points |
| --- | --- | --- | --- | --- | --- | --- | --- | --- | --- |
| 1 | Gupta K | PCCM 2012  21283046 | Efficacy of daily interruption of continuous sedative infusion to reduce ventilation | RCT  unblinded | Single-center PICU | January 2007-December 2007/ North India | 102 Pts/ age mean 4.1 years  Mechanically Ventilated for >48 hours | To compare the length of MV and duration of PICU stay | Number and % of days awake on sedative infusion, AEs, total dose of sedatives |
| 2 | Verlaat CWM | Paediatr Anaesth 2014  23980693 | Feasibility of daily interruption of sedatives in critically ill children | RCT  unblinded | Single-center PICU | November 2004-October 2006/The Netherlands | 30 Pts / 0-12 years  Mechanically Ventilated for >24 hours | To determine the total amount of sedatives and number of bolus in the first 3 days, the number of incidents | Length of MV and duration of PICU stay, changes in COMFORT-B |
| 3 | Curley MAQ | JAMA 2015  25602358 | Protocolized sedation in PICU: nurse-implemented, goal-directed sedation protocol | RCT  Cluster randomized design  unblinded  RESTORE study | Multi-centers  31 PICUs | June 2009-December 2013/USA | 2449 Pts /2 weeks-17 years  Pts mechanically ventilated for acute respiratory failure | To determine the impact of protocolized sedation on the duration of MV | Time to recovery from respiratory failure, weaning from MV, PICU and hospital LOS, mortality, sedation-related AE, IWS occurrence |
| 4 | Vet NJ | ICM 2016  26602782 | Combined use of daily sedation interruption and protocolized sedation | RCT  blinded | Multi-centers  3 PICUs | October 2009-August 2014/ The Netherlands | 129 Pts/0-18 years  Critically ill children mechanically ventilated for at least 48 h | Number of ventilator-free days at day 28: number of days without MV (continuously ≥48 h) during 28 days period after randomization | PICU and hospital LOS 30-day mortality, sedatives total doses, additional sedative drugs, number COMFOT-B <11 and>22, incidence of IWS. AEs |
| 5 | Franck LS | PCCM 2008  18838937 | Assessment IWS in pediatric PICU patients | Prospective observational with repeated-measures design. | Two PICUs | February 2004-April 2006/USA | 83 Pts/2 weeks-18 years, median 35 months  Pts with acute respiratory failure received continuous opioids infusion >5 days | To develop and test the validity and reliability of WAT-1 score |  |
| 6 | Ista E | CCM 2008  18596622 | Benzodiazepine and opioid withdrawal | Prospective observational with repeated-measures design. | Two PICUs | September 2005-February 2006/The Netherlands | 79 Pts/0-16 years, median 3.4 months  Pts received intravenous MDZ and/or opioids for ≥ 5 days | To evaluate the frequencies of withdrawal symptoms and form the basis for an assessment tool | Correlations with total doses and duration |
| 7 | Ista E | PCCM 2013  23962832 | Assessment IWS in pediatric PICU patients | Prospective observational with repeated-measures design. | Single-center PICU | March 2009-September 2010/The Netherlands | 154 Pts/0-16 years, median 5 months  Pts received intravenous BDZ and/or opioids for ≥ 5 days | To establish SOS value, cutoff scores and test sensitivity to change | To explore risk factors for IWS |
| 8 | Best KM | PCCM 2016  26509816 | Analysis of patterns of sedation weaning among critically ill children recovering from acute respiratory failure | Secondary analysis of data prospectively collected during pre-randomization phase of RESTORE trial | Multi-centers  22 PICUs | January-July 2009/USA | 145 Pts/2 weeks-17 years.  Pts experienced ≥5 days of opioids during MV for acute respiratory failure | Descriptive analysis: Pts characteristics, presence of tolerance and IWS |  |
| 9 | da Silva PSL | J Addict Med 2016  26927302 | IWS: risk factor, rate, outcomes | Prospective Observational Study | Single-center PICU | January 2012-December 2014/Brazil | 137 Pts/1 month-16 years  Pts requiring MV ≥72 h and received continuous FNT-MDZ infusion ≥3 days | To evaluate the incidence of withdrawal syndrome in PICU Pts | Assessed risk factors and Pts outcome |
| 10 | Vet NJ | PCCM 2016  27662565 | Effect of daily sedation interruption + protocolized sedation  on short-term clinical outcome (8 weeks after PICU discharge) | Preplanned prospective analysis of RCT | Two PICUs | The Netherlands | 64 Pts/0 -18 years Critically ill children mechanically ventilated at least 48 h | To compare short-term health-related quality of life and posttraumatic stress symptoms between the two RCT groups  (see study n.4) |  |
| 11 | Best KM | CCM 2017  27513532 | Risk factor for IWS: identify the clinical profile of Pts, process and system-level factors, predictors of IWS | Secondary analysis  of the RESTORE (RCT) database | Multi-centers  31 PICUs | June 2009-December 2013/USA | 1157 Pts (47.2% of all RESTORE study PTS)/2 weeks-17 years. Pts with ≥5 days of sedation during MV for acute respiratory failure | To generate a predictive model of risk factors for IWS |  |
| 12 | Amigoni A | PCCM 2017  28157809 | Occurrence of IWS | Prospective Observational study | Multi-centers  8 PICUs | November 2012-May 2014/Italy | 113 Pts mechanically ventilated treated with analgesic and sedatives ≥5 days/<18 years | To establish the frequency of IWS in Italian PICUs | The difference in Pts clinical profile, sedatives treatments, outcome |
| 13 | Gaillard-Le Roux B | PCCM 2017  27801707 | Nurse-driven sedation protocol in a PICU | Two-phase prospective observational control study. Before (15 months) and after (18 months) protocol implementation | Single-center PICU | October 2010-December 2013/France | 235 Pts/ 28 days-18 years  Pts requiring MV for more than 24 h | To evaluate the impact of nurse-driven sedation protocol on MV | To evaluate the impact of a protocol on sedatives daily-doses, and sedation complications |
| 14 | Dreyfus L | Ann. Intensive Care 2017  28341980 | Nurse-driven sedation protocol in a PICU | Two-phase prospective observational control study. l Before (12 months) and after (11 months) protocol implementation | Single-center PICU | January 2013-March 2015/France | 200 Pts/ 0-18 years  Pts requiring MV for more than 24 h | To evaluate the impact of nurse-driven sedation protocol on MV | Impact of protocol on sedatives total doses and duration, PICU LOS, incidence of VAP, IWS |
| 15 | Neunhoeffer F | Eur J Pediatr Surg 2017  27454068 | Nurse-driven sedation protocol in a PICU | Two-phase prospective observational control study. Before (15 months) and after (15 months) protocol implementation | Single-center PICU | October 2010-March 2013/Germany | 226 Pts/1-16 years Mechanically Ventilated (>24 h) postsurgical children | To evaluate the impact of a protocol on MV duration, total doses of BDZ and opioids, PICU LOS, occurrence of WS | To investigate the effect in subgroups not naïve to BDZ or opiates (oncological, post-transplantation) |
| 16 | Mencía S | PCCM 2018  29557840 | Sevoflurane as an alternative for difficult sedation in PICU | Prospective Observational study | Two PICUs | June 2011-June 2017/Spain | 23 Pts/ 1mounth-16 years, median 6 months | Effectiveness of sevoflurane in critically ill children difficult to sedate |  |
| 17 | Sanavia E | PCCM 2019  31261229 | Drug rotation protocol | Prospective Observational study | Single-center PICU | June 2012—June 2016/Spain | 100 Pts/1 month-16 years  Pts requiring sedative-analgesic continuous infusion >4 days | To assess the implementation of sedative and analgesic drug rotation protocol | To analyze the incidence of WS, drug doses, time of drugs infusion |
| 18 | Pavcnik M | Minerva Anestesiol  2019  30871299 | Sevoflurane during weaning from MV | Prospective Observational study | Single-center PICU | January 2014-January 2017/Slovenia | 40 Pts/Pts ready for weaning from MV and with WAT-1 ≥8 at de-escalation of sedatives | To evaluate feasibility and efficacy of sevoflurane sedation during weaning from MV | Effects on hemodynamics, development of psychomotor dysfunction after discontinuation |

|  | Intervention/Method | Control Group/ Comparison group | Main Results | Measurements | Data Analysis | Strengths and limitations |
| --- | --- | --- | --- | --- | --- | --- |
| 1 | Interrupted sedative infusion  46 Pts | Continuous sedative infusion  56 Pts | Length of MV, duration of PICU stay and total dose of midazolam  were significantly reduced in the interrupted group.  AE were similar in both the groups | Total duration of MV and length of ICU stay. Awake days on sedation, AE, total dose of sedatives  Ramsay score | Comparison of variables by *t* test or Mann-Whitney *u* test. Survival analyses (Kaplan-Meier) and Cox proportional hazard regression analyses to adjust for confounding factors |  |
| 2 | Interrupted sedative infusion  15 Pts | Standard of care  15 Pts | The total amount of sedatives was lower in the intervention group. N° of bolus similar. Length of MV and duration of PICU stay were significantly reduced. No differences in incidents were found  COMFORT-B showed more variation and were higher in the intervention group | COMFORT-B | Comparison of variables by Mann-Whitney *u* test and Fishers exact test. Kaplan-Meier survival analysis with *P*-value calculated by Log-Rank test |  |
| 3 | Protocolized sedation  17 PICU,1225 Pts | Usual care  14 PICU,1224 Pts | Duration of MV was not different Sedation-related AE were not different. Intervention Pts were exposed to fewer days of opioids, fewer sedative classes. They were more often awake and calm while intubated and had more days without any report of pain or agitation | FLACC scale, Wong-Baker Faces Pain Scale, INR scale, SBS scale, WAT-1 scale | Comparison analysis by Kaplan-Meier curves and proportional hazards regression. Exploratory analysis by  logistic, multinomial logistic, cumulative logit, linear and Poisson regression |  |
| 4 | Daily sedation interruption + protocolized sedation  66 Pts | Protocolized sedation  63 Pts | The study was terminated prematurely due to slow recruitment rates.  The number of ventilator-free days does not differ. Median PICU and hospital LOS not differ. Median cumulative sedative drugs dose not differ. Undersedation was more frequent in the intervention group. Mortality at 30 days was higher in the intervention group | COMFORT-B, NISS, NRS, SOS | Comparison of variables by Mann-Whitney test and Fishers exact test. Multiple linear regression analysis. Kaplan-Meier survival analysis and log-rank test. Penalized Cox analysis | Downgraded in quality assessment due to the low recruitment rate than the planned number |
| 5 | None  Pediatric critical care nurses assessed eligible at-risk pediatric patients for the presence of 19 withdrawal symptoms and rated the patient’s overall withdrawal intensity using a Numeric Rating Scale |  | WAT-1 is an 11 items (12 points) scale. The scale showed good concurrent and construct validity for score >3 | 19 symptoms from Opioid and Benzodiazepine Withdrawal Score | Psychometric evaluation. Descriptive statistics and univariate analysis |  |
| 6 | None  Nurses assessed withdrawal symptoms using Sophia Benzodiazepine and Opioid Withdrawal Checklist (24 symptoms) |  | A significant correlation was found between total doses and duration of use and max sum score | 24 symptoms from Sophia Benzodiazepine and Opioid Withdrawal Checklist | Descriptive statistics, Spearman’s rank-correlation coefficient. Psychometric evaluation  Univariate analysis |  |
| 7 | None  Nurse’s assessment IWS using SOS and NRS withdrawal that represents the opinion of the severity |  | SOS is a valid tool suitable for BDZ and opioid WS; the cutoff score is ≥4; SOS shows sensitivity to change. Risk Factors for IWS are: duration of preweaning (especially MDZ), duration of weaning and number of additional sedatives/opioids | SOS and NRS withdrawal intensity rating | Multivariate analysis, Multilevel regression analysis |  |
| 8 | None |  | AA classified two patterns of weaning: intermitted and steady. Intermitted weaned PTS (n°66) received higher peak and cumulative doses of sedatives, longer exposures, had WAT-1 ≥3, received more rescue bolus and sedative classes than steadily weaned Pts (n°79) | WAT-1 | Descriptive statistics, comparison of variables. Logistic, multinomial logistic, cumulative logit, linear and Poisson regression |  |
| 9 |  |  | Incidence of IWS 22.6%. Of the 31 IWS, 6 received continuous sedatives infusion <5 days. Pts requiring MDZ above 0.35 mg/Kg/h are at risk for IWS. IWS prolonged MV, PICU stay, weaning of sedatives | SOS | Univariate analysis and multivariable analysis using logistic regression |  |
| 10 |  |  | No differences were found considering the health-related quality of life between groups. None Pts (>4 years) showed posttraumatic stress symptoms. No beneficial effects of daily sedation interruption | Child Health Questionnaire, Dutch Children’s Responses to Trauma Inventory (for age > 4 years) | Fisher exact test, Mann-Whitney tests. Kolmogorov-Smirnov test. One-sample Wilcoxon signed-rank test, paired Wilcoxon signed-rank test. |  |
| 11 | Opioids were weaned first with a 10% reduction every 8 h. Once opioids were discontinued, benzodiazepines were weaned with 20% dose reductions at least every 24 hours, with adjustments again based on WAT-1 scores | Weaning was left to provider discretion | 544/1157 (47%) Pts showed IWS (at least twice WAT-1 ≥3). Subjects with IWS were: younger (under 6 months), with preexisting cognitive impairment, had received higher sedative doses for more time, more sedatives classes, higher nursing workload, inadequate nurse staffing | SBS, WAT-1, Process-level factors: SBS assessment compliance. System-level factors: nurse-to-patient staffing ratio, NEMS (Nine Equivalents of Nursing Manpower Use Score) | Intraclass correlation coefficient (ICC) across PICU sites. Multivariate analysis, multivariate logistic regression |  |
| 12 | Symptoms of WS were monitored with WAT-1 scale |  | 73 (64.6%) Pts developed IWS (at least one WAT-1 ≥3). Pts with WS had a longer duration of analgosedation therapy, larger cumulative doses, longer duration of weaning, MV, PICU stay, and were rated as being difficult to treat. Lower incidence of WS in Pts who received morphine | WAT-1 | Multivariable logistic regression, stepwise logistic regression |  |
| 13 | Second phase: nurse-driven sedation Protocol. Two target sedative levels (COMFORT-B 11-17 or 7-11)  97 Pts | First phase: before protocol  97 Pts | Length of MV does not differ. In children > 12 months (subgroup analysis) length of MV was shorter. The daily dose of MDZ decreased. No differences were observed for secondary outcomes | COMFORT-B, SOS | Descriptive statistics, comparison of variables by chi-squared test, Kruskal-Wallis test. Kaplan-Meier survival Log-Rank test | Upgraded in quality assessment due to consistency |
| 14 | After protocol implementation  93 Pts | Before protocol  104 Pts | The number of optimal scores significantly increased. Duration of MV tended to be lower but the change was not significant. In the intervention group COMFORT-B scores per day increased. No differences were observed for secondary outcomes | COMFORT-B, WAT-1 | Comparison analysis controlled for age and reason for admission. Segmented regression analyses |  |
| 15 | After protocol implementation, Target level COMFORT-B 12-18,  NISS 2  110 Pts | Before protocol  116 Pts | PICU LOS or duration of MV did not differ. Total and daily doses of BDZ were significantly reduced, but not of opioids.  Compliance with the Scoring improved. Protocol compliance improved. The rate of IWS was significantly lower but not after solid organ transplantation or in oncological Pts | COMFORT-B, SOS, NISS | Comparison of variables by Student t-test, Wilcoxon rank-sum test, chi-square test. Subgroup analysis in oncologic surgical Pts (39 Pts) and solid organ transplantation (39 Pts) |  |
| 16 | Sevoflurane administered via AnaConDa plus morphine | Local protocol | BIS value decreased significantly. Median ETsevo 0.8%, median infusion time 5 days. In 78% after 48h of treatment, some sedatives could be removed. WS may occur after suspension | COMFORT scale, BIS, SOS | Descriptive statistics, comparison of variables by Mann-Whitney *u* test and Fishers exact test |  |
| 17 | sedative and analgesic drug rotation protocol adequately received | Protocol inadequately received | Compliance with the protocol: 35% of Pts received protocol correctly. No differences in the level of sedation or pain control. Significantly lower incidence of WS, shorter PCU LOS, shorter drugs infusion | COMFORT, Multidimensional Assessment Pain Scale, SOS, compliance with the protocol by auditing record sheets | Comparison of variables by chi-square test, Mann-Whitney *U* test. Kaplan-Meier survival analysis |  |
| 18 | After switching to sevoflurane sedation.  An anesthetic device placed in the inspiratory limb | Pre switching to sevoflurane sedation. An anesthetic device placed at Y-piece | Sevoflurane administration is technically feasible in PICU. Desired sedation was provided for 89% of the time. Mean arterial pressure decreased by 25%. Kidney and liver function no change.  Reversible psychomotor dysfunction developed in 12.5% | COMFORT, WAT-1, Bispectral Index (BIS) monitoring | Comparison of variables by Mann-Whitney test, Student’s *t*-test, Wilcoxon signed-rank test, Fisher exact test. Correlations by Spearman test |  |

Legend: AA: Authors; AE: Adverse Events; BDZ: Benzodiazepine; BIS: Bispectral Index Score; CCM: Critical Care Medicine; FNT: Fentanyl; ICM: Intensive Care Medicine; IWS: Iatrogenic Withdrawal Syndrome; LOS: length of stay; MDZ: Midazolam; MV: Mechanical Ventilation; NISS: Nurse Interpretation of Sedative Score; PCCM: Pediatric Critical Care Medicine; Pts: patients; RCT Randomized Controlled Trial; SBS: State Behavioral Scale; SOS: Sophia Observation withdrawal Symptoms; VAP: ventilator-associated pneumonia; WAT-1:Withdrawal Assessment Tool-1 scale
