# Supplementary material for: Recommendations for analgesia and sedation in critically ill children admitted to intensive care unit
Source: J Anesth Analg Crit Care. 2022 Feb 12;2:9. doi: 10.1186/s44158-022-00036-9 (PMC8853329; doi:10.1186/s44158-022-00036-9)
Supplement: Supplementary file 1 — Additional file 1. Synoptic Tables (files: Suppl Mat 1a, 1b, 1c, 1d, 1e, 1f, 1g, 1h). [file 44158_2022_36_MOESM1_ESM.zip › Additional file 1/JAACC Suppl Mat 1c NMBA .docx]

|  | First Author | Journal, Year,  PMID | Research Question | Design | Setting | Period (years)/Country | Patients/Age | Primary end-point | Secondary end-points |
| --- | --- | --- | --- | --- | --- | --- | --- | --- | --- |
| 1 | Weber F | Paediatr Anaesth. 2010  20670234 | How NMBA may affect hypnotic agents. Influence of muscle relaxants on BIS and Cerebral State Index (CSI) | RCT  blinded | Single center | March 2008-November 2008/ The Netherlands | 40 Pts/2-12 years  Pts scheduled for minor surgery | To detect the influence of muscle relaxant bolus on the BIS and CSI during propofol anesthesia |  |
| 2 | Janda M | Paediatr Anaesth. 2013  23910069 | The impact of muscle relaxants on stress response to endotracheal intubation in children | RCT  blinded | Single center | Germany | 38 Pts/2-6 years  Pts scheduled for adenotonsillectomy | To analyze the heart rate variability (HRV) to assess alterations in the sympathovagal balance |  |
| 3 | Chin KH | PCCM 2015  25599147 | Effect on NMBA in severe traumatic brain injury (TBI) on complication rates and outcomes | Secondary analysis from RCT  “Cool Kids Trial” | Multicenter  17 PICUs | USA, New Zealand, Australia | 90 Pts/< 18 years. Pts with severe TBI | To evaluate the association between NMBA and intracranial pressure (ICP), complication rates, and outcomes in children with TBI |  |
| 4 | Glau CL | PCCM 2018 29406380 | Degree of diaphragm atrophy in children on MV with and without NMBA | Prospective observational study | Single center | January 2016- April 2017/USA | 56 Pts/< 18 years. Pts with Acute Respiratory Failure MV for >24h | To evaluate the correlation between diaphragm thickening fraction and spontaneous breathing fraction and the exposure to continuous MNBA/corticosteroids |  |

|  | Intervention/Method | Control Group/ Comparison group | Main Results | Measurements | Data Analysis | Strengths and limitations |
| --- | --- | --- | --- | --- | --- | --- |
| 1 | Mivacurium group | Control group | Administration of mivacurium in Pts anesthetized with propofol has no impact on BIS and CSI values. | BIS, CSI | Mann-Whitney Rank Sum Test, paired t-test | Downgraded in quality assessment due to directness limitation |
| 2 | NMB group | NoNMB group | Endotracheal intubation without NMB induced stronger stress response.  No significant difference in HRV changes immediately after NMB/Placebo administration. BIS value comparable during intubation. Significantly higher low-frequency power and the low/high-frequency ratio immediately after endotracheal intubation compared before laryngoscopy in the No-NMB group. It is hard to rate the clinical relevance of stress reduction when using NMBA | Frequency domain analysis of HRV, RR intervals, BIS | Chi-square test, Mann-Whitney *U*-test | Downgraded in quality assessment due to directness limitation: low generalizability to PICU setting |
| 3 |  |  | Administration of NMBA was associated with an increase in ICP-related therapies. NMBA was not associated with mortality/outcome effect or increased incidence of complications (pneumonia, infections, hypotension, and seizures). NMBA usage was associated with prolonged ICU LOS | ICP, CPP, mPILOT score, GOS-E Peds | Mann-Whitney U test, Pearson *X*^2^ statistic with Yates’ continuity correction, Fisher’s exact test, Kaplan-Meier survival analysis, log-rank test. Regression model |  |
| 4 |  |  | Median age 17 months, the median duration of MV 140 h. The change in thickness at end-expiration between the first and last measurement was -13.8%. Thickness fraction was significantly associated with spontaneous breathing fraction. Pts exposure to NMBA had significantly a decrease in thickness at end-expiration. Extubation failure occurred in 3.6% and 44.6% required NIV. Greater Diaphragm Atrophy was observed in Pts exposed to corticosteroids during MV. | Diaphragm thickness at end-expiration and end-inspiration by ultrasound within 36 h of intubation and pre-extubation. MV duration, mode of ventilation, spontaneous breathing fraction. Extubation success and NIV after extubation | Wilcoxon sign-rank test. Wilcoxon rank-sum test. Linear regression |  |

Legend: BIS: Bispectral index; CCP: Cerebral Perfusion Pressure; GOS-E Peds: Glasgow Outcome Score-Extended for Pediatrics; ICP: intracranial pressure; mPILOT: modified Pediatric Intensity Level of Therapy; MV: Mechanical Ventilated; NIV: non-invasive ventilation; NMBA: neuromuscular blocking agents;
