# Supplementary material for: Recommendations for analgesia and sedation in critically ill children admitted to intensive care unit
Source: J Anesth Analg Crit Care. 2022 Feb 12;2:9. doi: 10.1186/s44158-022-00036-9 (PMC8853329; doi:10.1186/s44158-022-00036-9)
Supplement: Supplementary file 1 — Additional file 1. Synoptic Tables (files: Suppl Mat 1a, 1b, 1c, 1d, 1e, 1f, 1g, 1h). [file 44158_2022_36_MOESM1_ESM.zip › Additional file 1/JAACC Suppl Mat 1d Sleep.docx]

|  | First Author | Journal, Year,  PMID | Research Question | Design | Setting | Period (years)/Country | Patients/Age | Primary end-point | Secondary end-points |
| --- | --- | --- | --- | --- | --- | --- | --- | --- | --- |
| 1 | Armour A | J Burn Care Res 2008  18182928 | Sleep enhancing interventions | RCT  Blinded crossover design | Single-center PICU | USA | 40 Pts/ 3-18 years  Burn Pts (TBSA >20%) | To evaluate the effects of 2 sleep-inducing treatments (zolpidem and haloperidol) on sleep architecture in pediatric burn Pts | To examine factors associated with higher symptom scores |
| 2 | Gottschlich MM | J Burn Care Rehabil. 1994  7852451 | Sleep after burn injury | Prospective Observational Study | Single-center PICU | May 1991-September 1993/USA | 11Pts/1.4-16 years  Burn Pts (TBSA >/=20%) | To characterize sleep pattern of burn Pts |  |
| 3 | Al-Samsam RH | PCCM 2005 16148818 | Sleep pattern in PICU Pts MV. Effect of noise and staff interventions on sleep pattern | Prospective Observational Study | Single-center PICU | September 2000- June 2001/UK | 11 Pts/3-21 months. Intubated, MV, sedated with morphine and midazolam | The architecture of sleep in PICU Pts MV | To investigate the effect of noise and staff interventions on sleep pattern |
| 4 | Gottschlich MM | J Burn Care Res 2011  21912336 | Relationship of medications (ketamine) to sleep architecture | A subanalysis of data collected during the Armour RCT | Single-center PICU | USA | 40 Pts/3-18 years  Burn Pts (TBSA >/=20%) | To describe the effect of ketamine on sleep patterns in burn Pts |  |
| 5 | Armour A | J Burn Care Res 2011  21912335 | Assessment of sleep among pediatric burn Pts | Secondary analysis of observational data prospectively collected by Armour RCT | Single-center PICU | USA | 40 Pts/ 3-18 years  Burn Pts (TBSA >20%) | To compare observational visual assessment with to PSG in the determination of sleep quality |  |
| 6 | Benissa MR | J Clin Monit Comput 2016  26515742 | Assess sleep stages |  |  | March 2012-November 2012/France | 12Pts referred for sleep-disordered breathing/5-30 years | To correlate BIS values with PSG sleep stages to determine BIS thresholds to discriminate sleep stages | To determine the effect of the different signal quality index thresholds on BIS data interpretation |

|  | Intervention/Method | Control Group/ Comparison group | Main Results | Measurements | Data Analysis | Strengths and limitations |
| --- | --- | --- | --- | --- | --- | --- |
| 1 | Each Pt alternately received zolpidem (2 nights) one week (during the second postburn week) and haloperidol (2 nights) the next week (during the third postburn week) | Control drug-free night (the night before) | Pediatric burn Pts show significant sleep deprivation  Zolpidem: increased (minimally)the proportion of ¾ and rapid eye movement sleep but not total sleep time. Haloperidol: increased (significantly) total sleep time and stage 2 sleep compared with control nights. There were no statistically significant differences between the drugs. To justify their use in this Pts research into more effective dosing is required | PSG recordings | *X*^2^, *t-*test, Wilcoxon’s rank-sum test. Mixed model analysis |  |
| 2 |  |  | Large aberrations in sleep stage distribution were noted: decreases in stage 3 and 4 and REM and increases in the stage 1 and 2. The mean total sleep time was seemingly adequate. | PSG recordings and observational subjective data | Multiple regression analysis |  |
| 3 |  |  | Noise levels >48 dB(A), night peak 103 dB(A). Staff’s interventions lasted mean of 240 mins in a 24-hr period, no difference in number between day and night. Active sleep or REM sleep was reduced to a mean of 3%, frequent awakening and severe sleep fragmentation. | Noise dosimeter and Quest Noise Manager software package. Duration and number of staff’s interventions. PSG | Two-tailed Student’s test, one-way analysis of variance |  |
| 4 | 23 Pts received ketamine | 17Pts non-ketamine group | Ketamine administration was associated with reduced REM sleep. Both groups were clearly REM deficient when compared with non-burden norms | PSG recordings | Chi-square test and *t*-test. Mixed model |  |
| 5 |  |  | The correlation between observation and PSG was poor: Pts were judged to be awake 9% of the time by observers and 52.3% of the time by PSG recordings. | PSG recordings and observational (awake, drowsy, asleep) assessments | Intraclass correlation (ICC) and weighted Kappa |  |
| 6 |  |  | BIS failed to distinguish pre-sleep wakefulness, wake after sleep onset, stage 1 and REM. BIS threshold that identified stage 2 was <73; stage 3 was <55 | PSG, BIS | Kruskal and Wallis ANOVA test, Dunn’s method for multiple comparisons. ROC curves were plotted to discriminate sleep stages from each other |  |

Legend: BIS: Bispectral Index; MV: Mechanical Ventilated; PSG: Polysomnography; REM: Rapid Eye Movement; ROC: Receiver Operating Characteristic; TBSA: Total Burn Surface Area;
