# Supplementary material for: Recommendations for analgesia and sedation in critically ill children admitted to intensive care unit
Source: J Anesth Analg Crit Care. 2022 Feb 12;2:9. doi: 10.1186/s44158-022-00036-9 (PMC8853329; doi:10.1186/s44158-022-00036-9)
Supplement: Supplementary file 1 — Additional file 1. Synoptic Tables (files: Suppl Mat 1a, 1b, 1c, 1d, 1e, 1f, 1g, 1h). [file 44158_2022_36_MOESM1_ESM.zip › Additional file 1/JAACC Suppl Mat 1e Delirium .docx]

|  | First Author | Journal, Year,  PMID | Research Question | Design | Setting | Period (years)/Country | Patients/Age | Primary end-point | Secondary end-points |
| --- | --- | --- | --- | --- | --- | --- | --- | --- | --- |
| 1 | Schieveld JNM | CCM 2008  18496355 | Pediatric delirium and pediatric mortality scoring systems values | Prospective Observational Study | Single-center PICU | January 2002-December2005/The Netherlands | 877 Pts /mean age 4.4 years | To study the predictive power of PIM and PRISM II about the occurrence of delirium | To examine the predictive power of PIM and PRISM II in relation to MV |
| 2 | Smeets IAP | Eur Child Adolesc Psychiatry 2010  19784857 | Impact of pediatric delirium on PICU LOS and financial costs | Prospective Observational Study | Single-center PICU | January 2002-February 2007/The Netherlands | 147 Pts (49 with delirium)/1-18 years | Association between delirium and PICU-LOS | Association between delirium and costs |
| 3 | Smith HAB | CCM 2011  20959783 | Delirium assessment:  pCAM-ICU | Prospective observational study | Single-center PICU | July 2008-March 2009/USA | 68 Pts (146 paired assessments)/$\geq$ 5 years | To validate the pCAM-ICU |  |
| 4 | Traube C | CCM 2014  24145848 | Delirium assessment:  CAPD | Prospective observational study | Single-center PICU | March-May 2012/USA | 111 Pts (248 paired assessments) /0-21 years | To validate the CAPD | To explore CAPD in subgroups (developmentally delay, respiratory support, prematurity, severity of illness) |
| 5 | Silver G | PCCM 2015  2564724 | Pediatric delirium and  associated risk factor | Secondary analysis of data from a prospective validation study of CAPD | Single-center PICU | Ten weeks/USA | 99 Pts/0-21 years | Delirium prevalence | Risk factor |
| 6 | Traube C | CCM 2016  27518377 | Cost associated with delirium in PICU | Prospective Observational Study | Single-center PICU | September 2014-December 2014/USA | 464 Pts/ | Cost associated with delirium in PICU |  |
| 7 | Smith HAB | CCM2016  26565631 | Delirium assessment:  PreSchool CAM-ICU | Prospective observational study | Single-center PICU | March 2013-October 2014/USA | 300 Pts (530 paired assessments)/6 months-5 years | To validate the PreSchool CAM-ICU | To determine delirium prevalence |
| 8 | Traube C | CCM 2017  28288026 | Epidemiology and outcomes (mortality) of delirium in PICU | Prospective Observational Longitudinal Cohort Study | Single-center PICU | September 2014-August 2015/USA | 1547 Pts (267 with delirium)/ 59% <5 years | Incidence, time to onset, duration, risk factors, outcomes | Determination of delirium subtype. Effect on the duration of MV and hospital LOS |
| 9 | Traube C | CCM 2017  28079605 | Point Prevalence of delirium in PICU | Prospective Observational Study | Multi-centers  25 PICUs | Two designated study days/USA, the Netherland, New Zealand, Australia, Saudi Arabia | 994 Pts (835 Pts evaluated) | To determine the prevalence of delirium in PICUs, Risk factors | To establish the practicality of bedside delirium screening (CAPD) |
| 10 | Meyburg J | PCCM 2017  27776085 | Postoperative delirium pediatric patterns | Prospective Observational Study | Single-center PICU | May 2014-October2014 /Germany | 93 Pts/0-17 years | Time course (shortly or longer) of delirium in children after elective surgery | Risk factor and outcome |
| 11 | Patel AK | PCCM 2017  27977539 | Pediatric post-cardiac bypass surgery delirium | Prospective Observational Study | Single-center PICU | December 2014-August 2015/USA | 194 Pts/one day-21 years  PCICU Pts following  Cardiac bypass surgery | Incidence of pediatric delirium after cardiac bypass surgery. Risk factor | Outcomes: PCICU LSO, time on MV |
| 12 | Smith HAB | CCM 2017  28594681 | Pediatric delirium in critically ill infants and young children, modifiable risk factors (BDZ) and short-term outcomes (PICU LOS) | Secondary analysis of data from a prospective validation study of the preschool CAM-ICU | Single-center PICU | March 2013-October 2014/USA | 300 Pts/6 months-5 years (preschool-age) | Association between delirium and modifiable risk factors (BDZ). Short-term outcomes: PICU-LOS |  |
| 13 | Simone S | PCCM 2017  28410275 | ICU bundle on delirium screening, prevention, treatment | Prospective observational study  Quality improvement project | Single-center PICU | December 2013-September 2015/USA | 1875 Pts (140 with delirium, 41 with emerging delirium) | To examine ICU bundle impact on delirium prevalence and adherence to CAPD-(R) | To describe characteristics of delirium |
| 14 | Alvarez RV | J Pediatr 2018  29395177 | Delirium in Cardiac PICU: incidence, risk factors, characteristics, short-term outcome | Prospective Observational Cohort Study | Single-center PICU | June 2015-August 2015/USA | 99 Pts/ <21 years | Incidence of delirium | Course of delirium, risk factors, outcomes |
| 15 | Slooff VD | PCCM 2017  2923997 | Delirium treatment: haloperidol and adverse events | Prospective observational study of a clinical dose-titrating protocol | Single-center PICU | July 2014-June 2015/The Netherlands | 13 Pts / median age 8.3 years (0.4-13.8 years) | To report safety and efficacy of haloperidol after implementation of a dosing protocol |  |
| 16 | Ista E | Aust Crit Care 2018  28843537 | Delirium assessment:  SOS-PD  An integrated tool to capture both IWS and Pediatric Delirium | Prospective observational study | Single-center PICU | April 2012- October 2013/ The Netherlands | 146 Pts (2088 assessments)/$\geq$3 months-16 years admitted to the PICU for >48 h | To determine the measurement properties of the PD-component of the SOS-PD |  |
| 17 | Ista E | Crit Care 2018  30458826 | Delirium assessment:  SOS-PD | Prospective observational study | Multi-centers  4 PICUs | December 2015-August 2016/ The Netherlands | 485 Pts (5207 assessments /$\geq$3 months-18 years admitted to the PICU for >48 h | To determine construct-validity, criterion validity, interrater reliability of the PD part of the SOS-PD | To assess the false-negative rate by a psychiatrist in a randomly selected group |
| 18 | Meyburg J | PCCM 2018  30059477 | Postoperative pediatric delirium risk factors | Prospective observational study | Single-center PICU | May 2014-October 2014/Germany | 93 Pts /0-17 years | To determine and quantify risk factors for delirium after major elective surgery |  |
| 19 | Ramirez CR | Med Intensiva 2019  29530328 | Delirium characteristics, prevalence, and risk factors in children $\geq5 years$ | Prospective, cross-sectional observational study | Single-center PICU | One year study period/Colombia | 156 Pts/5-14 years | To evaluate prevalence and factors associated with delirium in Pts between 5 and 14 years over 1 year period |  |

|  | Intervention/Method | Control Group/ Comparison group | Main Results | Measurements | Data Analysis | Strengths and limitations |
| --- | --- | --- | --- | --- | --- | --- |
| 1 |  |  | Pediatric illness severity measures (PIM and PRISM II) above the 60^th^ centile were associated with risk of delirium. Low positive predictive value and high negative predictive value.  The incidence of delirium was 4.5%. Risk Factor: MV. | PIM, PRISM II, DSM criteria for delirium diagnosis | Construction of ROC curves and identification of cut-off points. Calculation of the risk ratios | Upgraded in quality assessment due to low imprecision, low indirectness and low inconsistency |
| 2 |  |  | Pediatric delirium correcting for other prognostic factors prolonged (2.39 days) PICU LOS, and increased (1.5%) direct medical costs | Assessment by child neuropsychiatrist | *t* test, *X*^2^ tests. Linear regression model | Upgraded in quality assessment due to low imprecision, low indirectness and low inconsistency |
| 3 |  |  | The pCAM-ICU demonstrated a sensitivity of 83%, a specificity of 99% and high interrater reliability *k=* 0.96. It is a valid instrument for the diagnosis of pediatric delirium in children $\geq$ 5 years of age | pCAM-ICU, psychiatrist evaluation, RASS | Criterion validity, inter-observer reliability |  |
| 4 |  |  | The CAPD demonstrated a sensitivity of 94.1%, a specificity of 79.2%. Cronbach’s alpha 0.90, and high interrater reliability *k=* 0.94. In developmentally delayed children specificity was 51.2%, sensitivity 96.2%. Delirium prevalence was 20.6% | CAPD, psychiatrist evaluation, RASS | Criterion validity, inter-observer reliability |  |
| 5 |  |  | Delirium prevalence was 21%. Risk factors associated with delirium: age 2-5 years, MV, developmental delay. Hospital LOS was higher | DSM-IV criteria for delirium, RASS, | Comparison of variables by *t*-test, Wilcoxon rank-sum, chi-square test, Fischer exact test. Multivariable logistic regression, generalized estimating equation (GEE) analysis | Upgraded in quality assessment due to low imprecision, low indirectness and low inconsistency |
| 6 |  |  | Delirium occurrence 15.9%. Delirium increased (+ 85%) PICU stay cost. Cost increased incrementally with days spent delirious, this remained highly significant even after adjusting for covariates | CAPD | Kruskal-Wallis test to compare the total cost and average costs between groups. Multivariable linear regression |  |
| 7 |  |  | The PreSchool CAM-ICU short form demonstrated sensitivity 78%, specificity 86% and interrater reliability *k=* 0.79. Delirium prevalence was 44% using the PreSchool CAM-ICU more frequent in Pts younger than 2 years. Hypoactive delirium was predominant | PreSchool CAM-ICU, psychiatrist evaluation, RASS | Criterion validity, inter-observer reliability |  |
| 8 |  |  | The incidence of delirium was 17%. Duration 2 days (median). Delirium onset first 3 days. Prevalence of hypoactive and mixed subtypes. Risk factors: young age, developmental delay, MV, the severity of illness. Modifiable risk factors: deep sedation, BDZ, anticholinergics. Delirium was an independent predictor of mortality | CAPD, RASS | Comparison of variables by chi-square, Fischer exact test. Multivariable logistic regression, multivariable linear regression | Upgraded in quality assessment due to low imprecision, low indirectness and low inconsistency |
| 9 |  |  | Point prevalence was 25%, median prevalence was 23.3%. Risk factors independently associated  with delirium included age less than 2 years,  MV, benzodiazepines, narcotics, use of physical  restraints, and exposure to vasopressors and antiepileptics. Prevalence increased with length of time in the PICU. Delirium screening by the bedside nurse was feasible in children of all ages | CAPD, RASS | Univariate and multivariate analyses.  Comparison of variables by Fischer exact test, Wilcoxon signed rank-tests. Multivariate logistic regression | Upgraded in quality assessment due to low imprecision, low indirectness and low inconsistency |
| 10 |  |  | Delirium prevalence was 65.6 %. Half Pts with delirium were significantly younger Two different patterns: half Pts had short-lasting delirium and half longer lasting. Delirium was an independent predictor of increased Hospital LOS | CAPD, RASS | Comparison of variables by  *t*-test, chi-square test. Analysis of variance, analysis of covariance |  |
| 11 |  |  | Cardiac bypass surgery significantly increases pediatric delirium. The incidence of delirium in this sample was 49%. Most often delirium onset early first 1-3 days and lasting 1-2 days. Independent risk factors: age< 2 years, developmental delay, higher RACHS-1 score, cyanotic disease, albumin <3mg/dL. Delirium was an independent predictor of prolonged PICU LOS | CAPD, RASS | Comparison of variables by *t*-test, Wilcoxon rank-sum, chi-square test, Fischer exact test. Multivariate logistic regression. Multivariable linear regression |  |
| 12 |  |  | Prevalence of delirium 44%. Delirium was associated with a lower likelihood of ICU discharge. Greater BDZ was associated with increased risk and longer duration of delirium and a lower likelihood of ICU discharge | psCAM-ICU, RASS | Multivariable Cox proportional hazards regression models, negative binomial regression, multinomial logistic regression | Upgraded in quality assessment due to low imprecision, low indirectness and low inconsistency |
| 13 |  |  | Mean delirium rate decreased with the implementation of 3 PICU bundles of delirium, sedation, and early mobilization protocols | CAPD-R revised | Chi-square, Fisher exact tests, Kendall tau-b, Mann-Whitney *U* test. Statistical Process Control *U*-chart. Simple linear regression |  |
| 14 |  |  | Incidence was 57%. Delirium onset first 2 days. Risk Factor: young age, STS-EACTS longer MV, longer cardiopulmonary bypass time, BDZ.  Ondansetron decreased (81%) the odds of delirium.  Pts with delirium had worse outcomes, longer MV and LOS. In this cohort hypoactive delirium was most common | CAPD (each 12 h shift), RASS, WAT-1, | Comparison of variables by *t*-test, *X*^2^ tests, Wilcoxon rank-sum tests. Kaplan-Meier method. Multivariable mixed logistic regression model. Surgical subgroup (88 Pts) analysis was made |  |
| 15 | Treatment with haloperidol according to a dose-titration protocol |  | Adverse events (extrapyramidal symptoms and excessive sedation) developed despite low plasma concentrations ($\leq2.0microg/L)$. Range of IV dose 0.005-0.085 mg/Kg/d | SOS-PD scale, psychiatrist evaluation, haloperidol plasma concentration, screening for Aes: ECG, mSAS score | Because of the small cohort, no statistical analysis was performed to compare Pts with and without AEs.  Jonckheere-Terpstra test |  |
| 16 |  |  | Sensitivity 96.8%, specificity 92%, Intraclass Correlation Coefficient ICC 0.90. The scale shows promising validity for early screening of PD | SOS-PD, psychiatrist evaluation, CAPD, Comfort B, NISS | Chi-square tests, Mann-Whitney tests, linear mixed model. Inter-observer reliability, criterion validity, construct validity |  |
| 17 |  |  | The PD-scale has good reliability and validity for early screening of PD with sensitivity 92.3%, specificity 96.5%, Intraclass Correlation Coefficient ICC 0.99. Pearson coefficient for correlation between PD-scale and CAPD was 0.89. Delirium prevalence was 10% | SOS-PD, psychiatrist evaluation, CAPD, Comfort B, RASS | Chi-square tests, Mann-Whitney tests. Inter-observer reliability, criterion validity, construct validity | Upgraded in quality assessment due to low imprecision |
| 18 |  |  | The 66% of Pts were delirious. Risk factors: younger age, invasive catheters, respiratory devices, infections, type (inhalational) of anesthesia but not duration, cumulative doses of long-acting sedatives and analgesics, anticholinergics. Outcomes: longer duration of MV | CAPD | Pearson correlation, analysis of variance, chi-square test with Bonferroni correction. Analysis of covariance (ANCOVA). Multifactorial ordinal logistic regression analysis. |  |
| 19 |  |  | Delirium prevalence was 18.6%, predominantly (55.2%) hypoactive. Risk Factors of delirium were: intellectual disability, MV, liver failure, tachycardia, anticholinergic drug use, psychotropic agents, neurological disease | pCAM-ICU, Delirium Rating Scale Revised-98 (DRS-R98) for evaluation of motor function | Chi-squared test (*x^2^*), Mann-Whitney *U*-test (2 variables) or analysis of variance (ANOVA) with the Kruskal--Wallis test (more than 2 variables). Multivariate logistic analysis  Logistic regression analysis. Hosmer and Lemeshow goodness of fit test |  |

Legend: Aes: Adverse Events; BDZ: benzodiazepine; Dex: dexmedetomidine; CAPD: Cornell Assessment of Pediatric Delirium; CAM-ICU: Confusion Assessment Method for the ICU; ECG: electrocardiogram; IWS: Iatrogenic Withdrawal Syndrome; LOS: Length of stay; mSAS: modified Simpson Angus Scale; MV: Mechanical Ventilation; NISS: Nurse Interpretation Sedation Score; PCCM: Pediatric Critical Care Medicine; PCPC: Pediatric Cerebral Performance Category; Pts: patients; PIM: Pediatric Index of Mortality; PRISM II: Pediatric Risk of Mortality; pCAM-ICU: pediatric CAM-ICU; psCAM-ICU: preschool CAM-ICU; RASS: Richmond Agitation Sedation Scale; RCT: Randomized Controlled Trial; SOS-PD: Sophia Observation withdrawal Symptoms-Pediatric Delirium; STS-EACTS: Society of Thoracic Surgeons and the European Association for Cardiothoracic Surgery Congenital Heart Surgery Mortality Score; WAT-1: Withdrawal Assessment Tool-version1
