# Supplementary material for: Recommendations for analgesia and sedation in critically ill children admitted to intensive care unit
Source: J Anesth Analg Crit Care. 2022 Feb 12;2:9. doi: 10.1186/s44158-022-00036-9 (PMC8853329; doi:10.1186/s44158-022-00036-9)
Supplement: Supplementary file 1 — Additional file 1. Synoptic Tables (files: Suppl Mat 1a, 1b, 1c, 1d, 1e, 1f, 1g, 1h). [file 44158_2022_36_MOESM1_ESM.zip › Additional file 1/JAACC Suppl Mat 1f Withdrawal Syndrome.docx]

|  | First Author | Journal, Year,  PMID | Research Question | Design | Setting | Period (years)/Country | Patients/Age | Primary end-point | Secondary end-points |
| --- | --- | --- | --- | --- | --- | --- | --- | --- | --- |
| 1 | Ista E | CCM 2008  18596622 | Benzodiazepine and opioid withdrawal | Prospective observational with repeated-measures design. | Two PICUs | September 2005-February 2006/The Netherlands | 79 Pts/0-16 years, median 3.4 months  Pts received intravenous MDZ and/or opioids for ≥ 5 days | To evaluate the frequencies of withdrawal symptoms and form the basis for an assessment tool | Correlations with total doses and duration |
| 2 | Franck LS | PCCM 2008  18838937 | Assessment IWS in pediatric PICU patients | Prospective observational with repeated-measures design. | Two PICUs | February 2004-April 2006/USA | 83 Pts/2 weeks-18 years, median 35 months  Pts with acute respiratory failure received continuous opioids infusion >5 days | To develop and test the validity and reliability of WAT-1 score |  |
| 3 | Franck LS | Pain  2012  22093817 | Assessment IWS in pediatric PICU patients | Prospective observational with repeated measures. During pre-randomization phase of RESTORE study | Multi-centers  21 PICUs | January-July 2009/USA | 126 Pts/2 weeks-18 years median 1.6 years. Pts supported on MV for acute respiratory failure, exposed ≥5 days opioids | To evaluate psychometric properties and generalizability of WAT-1 |  |
| 4 | Ista E | PCCM 2013  23962832 | Assessment IWS in pediatric PICU patients | Prospective observational with repeated-measures design. | Single-center PICU | March 2009-September 2010/The Netherlands | 154 Pts/0-16 years, median 5 months  Pts received intravenous BDZ and/or opioids for ≥ 5 days | To establish SOS value, cutoff scores and test sensitivity to change | To explore risk factors for IWS |
| 5 | Amigoni A | 2014  25131427 | IWS: predictive factors | Prospective Observational study | Single-center PICU | October 2010-October 2011/Italy | 60 Pts/ 0-18 years. Pts treated with analgesic and sedatives ≥3 days | To evaluate the incidence of WS | Association between WS and factors related to analgesia and sedation treatment and Pts outcome. Association WAT-1-SOS-nurse judgment |
| 6 | Neunhoeffer F | Paediatr Anaesth 2015  25810086 | Nurse-driven sedation protocol in a PICU | Two-phase prospective observational control study. Before (15 months) and after (15 months) protocol implementation | Single-center PICU | October 2010-April 2013/Germany | 337 Pts/1-16 years critically ill non-surgical children Mechanically Ventilated, (>24 h) | To evaluate the impact of a protocol on the occurrence of WS | To evaluate the impact of a protocol on MV duration, total doses of BDZ and opioids, PICU LOS, |
| 7 | Best KM | PCCM 2016  26509816 | Analysis of patterns of sedation weaning among critically ill children recovering from acute respiratory failure | Secondary analysis of data prospectively collected during pre-randomization phase of RESTORE trial | Multi-centers  22 PICUs | January-July 2009/USA | 145 Pts/2 weeks-17 years.  Pts experienced ≥5 days of opioids during MV for acute respiratory failure | To characterize patterns of weaning and to compare different patterns of weaning |  |
| 8 | da Silva PSL | J Addict Med 2016  26927302 | IWS: risk factor, rate, outcomes | Prospective Observational Study | Single-center PICU | January 2012-December 2014/Brazil | 137 Pts/1 month-16 years  Pts requiring MV ≥72 h and received continuous FNT-MDZ infusion ≥3 days | To evaluate the incidence of WS in PICU Pts | Assessed risk factors and Pts outcome |
| 9 | Amigoni A | PCCM 2017  28157809 | Occurrence of IWS | Prospective Observational study | Multi-centers  8 PICUs | November 2012-May 2014/Italy | 113 Pts/<18 years Mechanically Ventilated treated with analgesic and sedatives ≥5 days | To establish the frequency of IWS in Italian PICUs | Difference in Pts clinical profile, sedatives treatments, outcome |
| 10 | Best KM | CCM 2017  27513532 | Risk factor for IWS: identify the clinical profile of Pts, process and system-level factors, predictors of IWS | Secondary analysis  of the RESTORE (RCT) database | Multi-centers  31 PICUs | June 2009-December 2013/USA | 1157 Pts (47.2% of all RESTORE study PTS)/2 weeks-17 years. Pts with ≥5 days of sedation during MV for acute respiratory failure | To generate a predictive model of risk factors for IWS |  |
| 11 | Neunhoeffer F | Eur J Pediatr Surg 2017  27454068 | Nurse-driven sedation protocol in a PICU | Two-phase prospective observational control study. Before (15 months) and after (15 months) protocol implementation | Single-center PICU | October 2010-March 2013/Germany | 226 Pts/1-16 years Mechanically Ventilated (>24 h) postsurgical children | To evaluate the impact of a protocol on MV duration, total doses of BDZ and opioids, PICU LOS, occurrence of WS | To investigate the effect in subgroups not naïve to BDZ or opiates (oncological, post-transplantation) |

|  | Intervention/Method | Control Group/ Comparison group | Main Results | Measurements | Data Analysis | Strengths and limitations |
| --- | --- | --- | --- | --- | --- | --- |
| 1 | None  Nurses assessed withdrawal symptoms using Sophia Benzodiazepine and Opioid Withdrawal Checklist (24 symptoms) |  | A significant correlation was found between total doses and duration of use and max sum score | 24 symptoms from Sophia Benzodiazepine and Opioid Withdrawal Checklist | Descriptive statistics, Spearman’s rank-correlation coefficient. Psychometric evaluation  Univariate analysis |  |
| 2 | None  Pediatric critical care nurses assessed eligible at-risk pediatric patients for the presence of 19 withdrawal symptoms and rated the patient’s overall withdrawal intensity using a Numeric Rating Scale |  | WAT-1 is an 11 items (12 points) scale. The scale showed good concurrent and construct validity for score >3 | 19 symptoms from Opioid and Benzodiazepine Withdrawal Score | Psychometric evaluation. Descriptive statistics and univariate analysis |  |
| 3 |  | WAT-1 <3 | Good psychometric performance and generalizability to assess WS. Pts with WAT-1 ≥3 showed greater cumulative opioid, longer length treatment and weaning compare to Pts with WAT-1 <3 | WAT-1 | Comparison of variables by van Elteren test, generalized linear mixed models, proportional hazards regression.  Psychometric evaluation: structural and construct validity | Upgraded in quality assessment due to directness |
| 4 | None  Nurses’ assessment IWS using SOS and NRS withdrawal that represents the opinion of the severity |  | SOS is a valid tool suitable for BDZ and opioid WS; cut-off score is ≥4; SOS shows sensitivity to change. Risk Factors for IWS are: duration of preweaning (especially MDZ), duration of weaning and number of additional sedatives/opioids | SOS and NRS withdrawal intensity rating | Multivariate analysis, Multilevel regression analysis |  |
| 5 | Blinded physician applied WAT-1. Study physician registered SOS and nurse’s judgment |  | The incidence of WS was 37%. Highest BDZ dose predicted the presence of WS, no other evaluated variables. Days of weaning were significantly higher in WAT-1 positive Pts. Outcome variables were not different. MDZ dose cut-off value 0.42 mg/Kg/h | WAT-1, SOS | Comparison of variables by: chi-square test, Fisher’s test, Wilcoxon test. Logistic regression analysis. ROC analysis to find cut-off for BDZ dose for the occurrence of WS |  |
| 6 | After protocol implementation. Target level COMFORT-B 12-18, NISS 2  172 Pts | Before protocol  165 Pts | Duration of MV and PICU LOS did not differ.  The total dose of BDZ and occurrence of WS were significantly reduced | COMFORT-B, SOS, NISS | Comparison of variables by Student t-test, Wilcoxon rank-sum test, chi-square test. |  |
| 7 | None |  | AA classified two patterns of weaning: intermitted and steady. Intermitted weaned Pts (n°66) received higher peak and cumulative doses of sedatives, longer exposures, had WAT-1 ≥3, received more rescue bolus of sedative classes than steadily weaned Pts (n°79) | WAT-1 | Descriptive statistics, comparison of variables. Logistic, cumulative logit, linear and proportional hazards regression |  |
| 8 |  |  | Incidence of IWS 22.6%. Of the 31 IWS, 6 received continuous sedatives infusion <5 days. Pts requiring MDZ above 0.35 mg/Kg/h are at risk for IWS. IWS prolonged MV, PICU stay, weaning of sedatives | SOS | Univariate analysis and multivariable analysis using logistic regression |  |
| 9 | Symptoms of WS were monitored with WAT-1 scale |  | 73 (64.6%) Pts developed IWS (at least one WAT-1 ≥3). Pts with WS had a longer duration of analgosedation therapy, larger cumulative doses, longer duration of weaning, MV, PICU stay, and were rated as being difficult to treat. Lower incidence of WS in Pts who received morphine | WAT-1 | Multivariable logistic regression, stepwise logistic regression |  |
| 10 | Opioids were weaned first with a 10% reduction every 8 h. Once opioids were discontinued, benzodiazepines were weaned with 20% dose reductions at least every 24 hours, with adjustments again based on WAT-1 scores | Weaning was left to provider discretion | 544/1157 (47%) Pts showed IWS (at least twice WAT-1 ≥3). Subjects with IWS were: younger (under 6 months), with preexisting cognitive impairment, had received higher sedative doses for more time, more sedatives classes, higher nursing workload, inadequate nurse staffing | SBS, WAT-1, Process-level factors: SBS assessment compliance. System-level factors: nurse-to-patient staffing ratio, NEMS (Nine Equivalents of Nursing Manpower Use Score) | Intraclass correlation coefficient (ICC) across sites. Multivariate analysis, multivariate logistic regression | Upgraded in quality assessment due to consistency and directness |
| 11 | After protocol implementation. Target level COMFORT-B 12-18,  NISS 2  110 Pts | Before protocol  116 Pts | PICU LOS or duration of MV did not differ. Total and daily doses of BDZ were significantly reduced, but not of opioids.  Compliance with the Scoring improved. Protocol compliance improved. The rate of IWS was significantly lower but not after solid organ transplantation or in oncological Pts | COMFORT-B, SOS, NISS | Comparison of variables by Student t-test, Wilcoxon rank-sum test, chi-square test. Subgroup analysis in oncologic surgical Pts (39 Pts) and solid organ transplantation (39 Pts) |  |

Legend: AA: Authors; AE: Adverse Events; BDZ: Benzodiazepine; BIS: Bispectral Index Score; CCM: Critical Care Medicine; FNT: Fentanyl; IWS: Iatrogenic Withdrawal Syndrome; LOS: length of stay; MDZ: Midazolam; MV: Mechanical Ventilation; NISS: Nurse Interpretation Sedation Scale; PCCM: Pediatric Critical Care Medicine; Pts: patients; RCT Randomized Controlled Trial; SBS: State Behavioral Scale; SOS: Sophia Observation withdrawal Symptoms; VAP: ventilator-associated pneumonia; WAT-1:Withdrawal Assessment Tool-1 scale; WS: Withdrawal Symptoms.
