# Supplementary material for: Recommendations for analgesia and sedation in critically ill children admitted to intensive care unit
Source: J Anesth Analg Crit Care. 2022 Feb 12;2:9. doi: 10.1186/s44158-022-00036-9 (PMC8853329; doi:10.1186/s44158-022-00036-9)
Supplement: Supplementary file 1 — Additional file 1. Synoptic Tables (files: Suppl Mat 1a, 1b, 1c, 1d, 1e, 1f, 1g, 1h). [file 44158_2022_36_MOESM1_ESM.zip › Additional file 1/JAACC Suppl Mat 1g Palliative Sedation .docx]

|  | First Author | Journal, Year,  PMID | Research Question | Design | Setting | Period (years)/Country | Patients/Age | Primary end-point | Secondary end-points |
| --- | --- | --- | --- | --- | --- | --- | --- | --- | --- |
| 1 | Wolfe J | J Clin Oncol 2015  25918277 | Patient-Reported Outcomes  (PROs) description of distress in children with advanced cancer | Prospective cohort study  (PediQUEST RCT) | Multi-center | 9 months of follow-up/USA | 104 Pts/2-18 years  Pts with advanced cancer | To describe symptom prevalence and distress. | To examine factors associated with higher symptom scores |

|  | Intervention/Method | Control Group/ Comparison group | Main Results | Measurements | Data Analysis | Strengths and limitations |
| --- | --- | --- | --- | --- | --- | --- |
| 1 |  |  | Children with advanced cancer experience high symptom distress: pain, fatigue, drowsiness, irritability, sleep disturbances. Factor associated with higher symptom scores: being female, brain tumor, disease progression and receiving intensive treatments. In subgroup analysis Pts who received mild cancer therapy at the end of life had lower scores compared with no therapy | PediQUEST Memorial Symptom Assessment Scale (PQ-MSAS) | Univariable analyses, linear mixed-effects models. Multivariable analyses. Subgroup analysis of Pts with end-of-life |  |

Legend: PediQUEST: Pediatric Quality of Life and Evaluation of Symptoms Technology in Children with Cancer;
