# Supplementary material for: Recommendations for analgesia and sedation in critically ill children admitted to intensive care unit
Source: J Anesth Analg Crit Care. 2022 Feb 12;2:9. doi: 10.1186/s44158-022-00036-9 (PMC8853329; doi:10.1186/s44158-022-00036-9)
Supplement: Supplementary file 1 — Additional file 1. Synoptic Tables (files: Suppl Mat 1a, 1b, 1c, 1d, 1e, 1f, 1g, 1h). [file 44158_2022_36_MOESM1_ESM.zip › Additional file 1/JAACC Suppl Mat 1h Neurodevelpmental Delay.docx]

|  | First Author | Journal, Year,  PMID | Research Question | Design | Setting | Period (years)/Country | Patients/Age | Primary end-point | Secondary end-points |
| --- | --- | --- | --- | --- | --- | --- | --- | --- | --- |
| 1 | Best KM | J Pediatr 2019  30527750 | Analgesia and sedation need in critically ill children with neurocognitive impairment | Secondary analysis  of the RESTORE (RCT) database | Multi-centers  31 PICUs | June 2009-December 2013/USA | 412 Pts (17% of all RESTORE study PTS)/media age 6.2 years. Pts with pre-existing cognitive impairment had baseline PCPC ≥3 | To compare analgesia and sedation management between critically ill children with pre-existing cognitive impairment and critically ill neurotypical children | To compare indicators of therapeutic efficacy |
| 2 | Valkenburk AJ | Anesth Analg 2009  19843782 | Sedation monitored by BIS in children who are intellectually disabled | Prospective observational study | Sigle center OR | September 2006-September 2007 | 17 pts intellectually disabled (2-13 years) scheduled for gastroduodenoscopy/PEG under general anesthesia vs 35 controls | To compare BIS values at different stages of anesthesia between intellectually disabled children and controls. | To investigate the discriminative properties of BIS between consciousness and unconsciousness for intellectually disabled children and controls. |

|  | Intervention/Method | Control Group/ Comparison group | Main Results | Measurements | Data Analysis | Strengths and limitations |
| --- | --- | --- | --- | --- | --- | --- |
| 1 | Analgesic and sedative drugs were prescribed according to protocol | Analgesic and sedative drugs were prescribed according to unrestricted usual care/local practice norms | Adjusting for age and severity of illness, cumulative doses of opioids and BDZ were lower in Pts with cognitive impairment, they had more study days awake and calm, and more documented iatrogenic withdrawal symptoms | FLACC scale, Wong-Baker FACES scale, INRS, SBS, WAT-1 | Multivariate statistics |  |
| 2 | BIS monitoring was performed during general anesthesia. Analgesic and sedative drugs were prescribed according to protocol | Analgesic and sedative drugs were prescribed according to protocol | Median BIS values for the intellectually disabled group were significantly lower than those for controls in the awake state, during stable intraoperative anesthesia, and during the return of consciousness. | Bispectral index values for both groups during different stages of anesthesia. | ROC curve | Small sample size |

Legend: BDZ: benzodiazepines; FLACC: Face, Legs, Activity, Cry, Consolability pain scale; ICM: Intensive Care Medicine; INRS: Individualized Numeric Rating Scale; PCCM: PCPC: Pediatric Cerebral Performance Category; Pts: patients; RCT: Randomized Controlled Trial; OR: Operatory Room; PEG: percutaneous endoscopy gastrostomy
