# Supplementary material for: Recommendations for analgesia and sedation in critically ill children admitted to intensive care unit
Source: J Anesth Analg Crit Care. 2022 Feb 12;2:9. doi: 10.1186/s44158-022-00036-9 (PMC8853329; doi:10.1186/s44158-022-00036-9)
Supplement: Supplementary file 3 — Additional file 3. Tables of Recommendations (file: Suppl Mat 3). [file 44158_2022_36_MOESM3_ESM.docx]

| **Recommendation 1** | |
| --- | --- |
| As a first-line strategy, we suggest optimizing analgesia using opiates and adopting alpha agonists as sedative agents, considering benzodiazepines a second-line. | |
| Level of evidence | C |
| Strength of recommendation | Moderate |
| Benefits | To avoid pain. Less risk of neurotoxicity and delirium due to the use of non-GABAergic drugs. |
| Harms and risks | Opiates may cause tolerance and withdrawal syndrome. Alfa agonists may promote haemodynamic negative effects. |
| Benefit-harm balance | Benefits overcome risks for the majority of patients. In patients with heart failure, the use of alfa agonists should be carefully evaluated. |
| Intentional vagueness (if applicable) | A specific molecule in the class of opiates to prefer is not specified |
| Value judgment | None |
| Exclusions (if applicable) | Patients not requiring a continuous analgesia sedation |
| Difference of opinion | None |
| Knowledge gaps and research opportunities | *Studies on weaning from a prolonged infusion of dexmedetomidine* |
| Presence of systemic review | *Vet et al 2013 Intensive Care Med (ref.13); Hayden et al 2016 Pediatr Crit Care Med (ref.30)* |

| **Recommendation 2** | |
| --- | --- |
| We suggest adopting protocols of analgesia and sedation to administer the minimal effective dose of analgesics and sedatives to reduce tolerance and the incidence of difficult analgesia/sedation. Furthermore, the daily interruption of sedation should be considered with caution. | |
| Level of evidence | C |
| Strength of recommendation | Moderate |
| Benefits | Reduction of withdrawal syndrome and duration of mechanical ventilation |
| Harms and risks | None |
| Benefit-harm balance | Benefits overcome risks |
| Intentional vagueness (if applicable) | Indication to adopt nurse-driven protocols |
| Value judgment | None |
| Exclusions (if applicable) | None |
| Difference of opinion | 6% of the panel doesn’t agree on the strength of recommendation |
| Knowledge gaps and research opportunities | *Studies on the efficacy of drug rotation (analgesics or sedatives) protocols* |
| Presence of systemic review | *None* |

| **Recommendation 3** | |
| --- | --- |
| We recommend regular monitoring with validated tools the level of analgosedation of pediatric patients admitted to Intensive Care Unit. | |
| Level of evidence | B |
| Strength of recommendation | Strong |
| Benefits | Evaluation of efficacy of analgesic and sedative therapy |
| Harms and risks | Risks are due to inaccurate monitoring. Increased workload. |
| Benefit-harm balance | Benefits overcome risks |
| Intentional vagueness (if applicable) | A specific validated scale to prefer is not specified |
| Value judgment | These tools may be not adequate in patients with neurodevelopmental delay. |
| Exclusions (if applicable) | Patients with neuromuscular blocking agents are excluded. |
| Difference of opinion | None |
| Knowledge gaps and research opportunities | *None* |
| Presence of systemic review | *None* |

| **Recommendation 4** | |
| --- | --- |
| In difficult analgesia/sedation we suggest using ketamine, due to its good safety profile. | |
| Level of evidence | D |
| Strength of recommendation | Weak |
| Benefits | Efficacy in guarantee an adequate level of sedation with a good safety profile |
| Harms and risks | Dissociative effect and drooling Possible neurotoxicity. |
| Benefit-harm balance | Benefits overcome risks particularly in patients with bronchospasm. |
| Intentional vagueness (if applicable) | None |
| Value judgment | None |
| Exclusions (if applicable) | Caution in patients with chronic cardiac insufficiency and in patients with arrythmias |
| Difference of opinion | 6% of the panel doesn’t agree on the strength of recommendation |
| Knowledge gaps and research opportunities | *Studies on the prolonged infusion of ketamine. Studies on efficacy and safety of other drugs used in difficult analgesia and sedation (i.e sevoflurane)* |
| Presence of systemic review | *None* |

| **Recommendation 5** | |
| --- | --- |
| We suggest using neuromuscular blocking agents in patients with severe respiratory insufficiency and persistent patient-ventilator asynchrony despite actions taken to limit the rate of asynchrony. | |
| Level of evidence | D |
| Strength of recommendation | Weak |
| Benefits | Reduction of ventilation trauma |
| Harms and risks | Reduction of diaphragmatic thickness |
| Benefit-harm balance | Benefits overcome risks in patients with severe respiratory insufficiency |
| Intentional vagueness (if applicable) | A specific molecule of neuromuscular blocking agent to prefer is not specified |
| Value judgment | None |
| Exclusions (if applicable) | Caution in patients with neuromuscular disease |
| Difference of opinion | None |
| Knowledge gaps and research opportunities | *Studies on length of treatment with*  *neuromuscular blocking agents* |
| Presence of systemic review | *None* |

| **Recommendation 6** | |
| --- | --- |
| We suggest monitoring the level of sedation with continuous processed EEG in patients treated with neuromuscular blocking agents, considering the limitation and the availability of the device. | |
| Level of evidence | D |
| Strength of recommendation | Weak |
| Benefits | To monitor the level of sedation if observational scales are not applicable |
| Harms and risks | Risks are due to inaccurate monitoring. Increased workload. |
| Benefit-harm balance | Benefits overcome risks |
| Intentional vagueness (if applicable) | A specific continuous processed EEG tool is not specified |
| Value judgment | None |
| Exclusions (if applicable) | It may be inaccurate in patients with status epilepticus and infants |
| Difference of opinion | 19% of the panel doesn’t agree on the strength of recommendation |
| Knowledge gaps and research opportunities | *Studies on continuous processed* *EEG in infants* |
| Presence of systemic review | *None* |

| **Recommendation 7** | |
| --- | --- |
| We recommend adopting in all pediatric patients admitted to Intensive Care Unit strategies to prevent sleep alterations, particularly non-pharmacologic ones (relaxing techniques, parental involvement, control of environmental factors). | |
| Level of evidence | X |
| Strength of recommendation | Strong |
| Benefits | To improve the quality of sleep impacts positively on patient’s outcome  and decreases the risk of delirium |
| Harms and risks | None |
| Benefit-harm balance | Benefits overcome risks |
| Intentional vagueness (if applicable) | None |
| Value judgment | Due to the paucity of literature, the search was conducted including older studies |
| Exclusions (if applicable) | None |
| Difference of opinion | *None* |
| Knowledge gaps and research opportunities | *Studies on prevention and treatment of sleep disorders in Pediatric Intensive Care Unit*  *Studies on monitoring the quality of sleep in in Pediatric Intensive Care Unit with tools other than polysomnography* |
| Presence of systemic review | *Kudchadkar et al 2014 Sleep Med Rev (ref.67); Lago et 2017 Acta Paediatr (ref.79)* |

| **Recommendation 8** | |
| --- | --- |
| We recommend working on modifiable risk factors of delirium, particularly reducing the use of benzodiazepines. | |
| Level of evidence | B |
| Strength of recommendation | Strong |
| Benefits | Reduction of the incidence of delirium ameliorate patient’s outcome |
| Harms and risks | None |
| Benefit-harm balance | Benefits overcome risks |
| Intentional vagueness (if applicable) | None |
| Value judgment | None |
| Exclusions (if applicable) | None |
| Difference of opinion | None |
| Knowledge gaps and research opportunities | *Studies on the efficacy of alpha agonists in reducing the incidence of delirium in critically ill patients.*  *Studies on the impact of reduction of modifiable factors in pediatric delirium.* |
| Presence of systemic review | *Daoud et al 2014 Crit Care (ref.81)* |

| **Recommendation 9** | |
| --- | --- |
| We suggest basing the treatment of pediatric delirium on maximizing preventive bundles. Antipsychotic drugs may be used with careful consideration of contraindications. | |
| Level of evidence | C |
| Strength of recommendation | Moderate |
| Benefits | Prevention development of delirium; treatment of delirium |
| Harms and risks | Risks are  due to antipsychotic drugs |
| Benefit-harm balance | Benefits overcome risks in the preventive phase. The benefit-harm balance of antipsychotic drugs needs to be considered |
| Intentional vagueness (if applicable) | A specific molecule of antipsychotic to prefer is not specified |
| Value judgment | Paucity of literature related to antipsychotic drugs in pediatric age |
| Exclusions (if applicable) | In patients with long QT trait and arrythmias antipsychotic treatment is not indicated |
| Difference of opinion | None |
| Knowledge gaps and research opportunities | *Studies on pharmacologic prevention and treatment of pediatric delirium* |
| Presence of systemic review | *None* |

| **Recommendation 10** | |
| --- | --- |
| We recommend regular monitoring delirium in critically ill children every day of the Intensive Care unit stay, using validated tools. | |
| Level of evidence | B |
| Strength of recommendation | Strong |
| Benefits | A prompt diagnosis permits a prompt therapy |
| Harms and risks | Risks are due to inaccurate monitoring. Increased workload. |
| Benefit-harm balance | Benefits overcome risks |
| Intentional vagueness (if applicable) | A specific tool to prefer is not specified |
| Value judgment | None |
| Exclusions (if applicable) | None |
| Difference of opinion | None |
| Knowledge gaps and research opportunities | *None* |
| Presence of systemic review | *None* |

| **Recommendation 11** | |
| --- | --- |
| We recommend working on modifiable risk factors of withdrawal synrdome, particularly avoiding weaning higher than a daily reduction of 20% respect on the initial dose. | |
| Level of evidence | B |
| Strength of recommendation | Strong |
| Benefits | Reduction of the incidence of withdrawal syndrome ameliorate patient’s outcome |
| Harms and risks | None |
| Benefit-harm balance | Benefits overcome risks |
| Intentional vagueness (if applicable) | None |
| Value judgment | Modality of weaning after the prolonged infusion is not detailed |
| Exclusions (if applicable) | None |
| Difference of opinion | None |
| Knowledge gaps and research opportunities | *Studies on weaning after ketamine and alpha agonists infusions* |
| Presence of systemic review | *Best et al Crit Care Med 2015 (ref. 118); Duceppe et al 2019 J Clin Pharmacol Ther (ref.119)* |

| **Recommendation 12** | |
| --- | --- |
| We recommend treating withdrawal symptoms with additional boluses of the drug considered to be responsible for the symptoms and modifying the weaning plan. | |
| Level of evidence | X |
| Strength of recommendation | Strong |
| Benefits | Treatment of withdrawal syndrome increases patient’s comfort and reduce correlated risks |
| Harms and risks | None |
| Benefit-harm balance | Benefits overcome risks |
| Intentional vagueness (if applicable) | The dose of the additional bolus is not specified |
| Value judgment | None |
| Exclusions (if applicable) | None |
| Difference of opinion | None |
| Knowledge gaps and research opportunities | *Studies on preventing withdrawal syndrome from adopting strategies of weaning or pharmacologic strategies* |
| Presence of systemic review | *Dervan et al 2017 Paediatr Anaesth (ref. 125)* |

| **Recommendation 13** | |
| --- | --- |
| We recommend regular monitoring withdrawal symptoms in critically ill children treated with analgesics and/or sedatives longer than 72 hours, adopting validated tools. | |
| Level of evidence | B |
| Strength of recommendation | Strong |
| Benefits | A prompt diagnosis permits a prompt therapy |
| Harms and risks | Risks are due to inaccurate monitoring. Increased workload. |
| Benefit-harm balance | Benefits overcome risks |
| Intentional vagueness (if applicable) | A specific tool to prefer is not specified |
| Value judgment | None |
| Exclusions (if applicable) | None |
| Difference of opinion | None |
| Knowledge gaps and research opportunities | *//* |
| Presence of systemic review | *None* |

| **Recommendation 14** | |
| --- | --- |
| We recommend performing pediatric palliative sedation early defining an interdisciplinary plan agreed with parents. | |
| Level of evidence | X |
| Strength of recommendation | Strong |
| Benefits | To enhance emotional and practical support for patients, family and staff |
| Harms and risks | None |
| Benefit-harm balance | Benefits overcome risks |
| Intentional vagueness (if applicable) | None |
| Value judgment | None |
| Exclusions (if applicable) | None |
| Difference of opinion | None |
| Knowledge gaps and research opportunities | *None* |
| Presence of systemic review | *None* |

| **Recommendation 15** | |
| --- | --- |
| We suggest adopting a personalized strategy to achieve pediatric palliative sedation in children, to ensure the maximal efficacy using doses tailored to the patient. | |
| Level of evidence | D |
| Strength of recommendation | Weak |
| Benefits | To guarantee comfort to incurable child during his/her last period of life |
| Harms and risks | None |
| Benefit-harm balance | Benefits overcome risks |
| Intentional vagueness (if applicable) | A molecule to prefer in pediatric palliative sedation is not indicated |
| Value judgment | None |
| Exclusions (if applicable) | None |
| Difference of opinion | 6% of the panel doesn’t agree on the strength of recommendation |
| Knowledge gaps and research opportunities | *Studies describing characteristics of pediatric palliative sedation in children in Intensive Care Unit.* |
| Presence of systemic review | *None* |

| **Recommendation 16** | |
| --- | --- |
| In children with developmental delay, we suggest adopting validated tools to monitor the level of sedation, the presence of delirium and withdrawal syndrome in Intensive care Unit, considering their limitations and involving the caregivers. | |
| Level of evidence | D |
| Strength of recommendation | Weak |
| Benefits | Obtain a prompt treatment of pain, discomfort, delirium and withdrawal symptoms |
| Harms and risks | Risks are due to inaccurate monitoring. Increased workload. |
| Benefit-harm balance | Benefits overcome risks |
| Intentional vagueness (if applicable) | None |
| Value judgment | A validated tool to evaluate discomfort, delirium and withdrawal symptoms in children with developmental delay doesn’t exist |
| Exclusions (if applicable) | None |
| Difference of opinion | None |
| Knowledge gaps and research opportunities | *Studies on pharmacological interactions between patient’s chronic therapy and analgesic and sedative treatment administered in Intensive Care Unit*  *Studies on the development of tools dedicated to children with developmental delay admitted to Intensive Care Unit* |
| Presence of systemic review | *None* |

| **Recommendation 17** | |
| --- | --- |
| We recommend explaining to parents the meaning of analgesia and sedation and off-label drugs. If analgesia and sedation lasted more than 48 hours, we recommend informing parents about the risk of withdrawal syndrome and delirium development. | |
| Level of evidence | X |
| Strength of recommendation | Strong |
| Benefits | Parents’ involvement in the plan of care |
| Harms and risks | Risks are related to a language barrier |
| Benefit-harm balance | Benefits overcome risks, particularly a good communication decrease the risk of conflicts |
| Intentional vagueness (if applicable) | None |
| Value judgment | None |
| Exclusions (if applicable) | None |
| Difference of opinion | None |
| Knowledge gaps and research opportunities | *//* |
| Presence of systemic review | *None* |
