# Supplementary material for: Recommendations for analgesia and sedation in critically ill children admitted to intensive care unit
Source: J Anesth Analg Crit Care. 2022 Feb 12;2:9. doi: 10.1186/s44158-022-00036-9 (PMC8853329; doi:10.1186/s44158-022-00036-9)
Supplement: Supplementary file 4 — Additional file 4. Questionnaire for caregivers (filer: Suppl Mat 4). [file 44158_2022_36_MOESM4_ESM.docx]

QUESTIONNAIRE FOR PARENTS ON ANALGESIA AND SEDATION OF CHILDREN ADMITTED TO ICU

Dear Mr/Mrs, we ask your collaboration to fill the present questionnaire with the aim to investigate parents’ opinions on the quality of analgesia and sedation administered to your son/daughter during the ICU stay. Based on your answers we will consider your contribution to increase children’s comfort in ICU in the future. We guarantee to you we will treat your responses confidentially.

***Thank you in advance for your help.***

1. Do you consider “good “the quality of information related to analgesia (pain control) and sedation administered to your son/daughter’s during the ICU stay? Some aspects were improvable? ________________________________________________________________________________________________________________________________________________________________________________________________________________________________
2. How do you consider the control of your son/daughter’s pain during ICU stay?________________________________________________________________________________________________________________________________________________________________________________________________________________________________
3. How do you consider the quality of your son/daughter’s sedation during ICU stay? _______________________________________________________________________________________________________________________________________________________________________________________________________________________________
4. Which factors have you considered disturbing and modifiable in your son/daughter’s environment? ___________________________________________________________________________________________________________________________________________________________________________________________________________________________________
5. Which suggestions may you give us to improve child’s comfort in ICU? _________________________________________________________________________________________________________________________________________________________________________________________________________________________________
6. In your opinion, have you adequately been informed about the risk of your son/daughter to develop withdrawal syndrome or delirium? _________________________________________________________________________________________________________________________________________________________________________________________________________________________________
