# Supplementary material for: Recommendations for analgesia and sedation in critically ill children admitted to intensive care unit
Source: J Anesth Analg Crit Care. 2022 Feb 12;2:9. doi: 10.1186/s44158-022-00036-9 (PMC8853329; doi:10.1186/s44158-022-00036-9)
Supplement: Supplementary file 5 — Additional file 5. Drugs (file: Suppl mat 5). [file 44158_2022_36_MOESM5_ESM.docx]

1. **Analgesics**

| **Molecule** | **Bolus dose** | **Continuous infusion dose** | **ONSET (min)** | **OFFSET (min)** | **Metabolism** | **Main side effects** | **OFF LABEL** |
| --- | --- | --- | --- | --- | --- | --- | --- |
| Fentanyl | 1-5 mcg/Kg | 1-6 mcg/kg/h | 1-2 | 30-60 | N-dealkylation liver hydroxylation  Inactive metabolites | Respiratory depression, hypertension, thoracic rigidity | Age< 2 years  see law 648/96 |
| Remifentanil | Age 1 months-11 years:  0.1-1mcg/Kg in 30 sec  Age ≥12 years:  0.1-1mcg/Kg in 30 sec | Age 1 mese-11aa:  3-78 mcg/Kg/h  Age >11 aa:  3-120 mcg/Kg/h | Rapid | Rapid, not dependent on dose and duration | Plasmatic esterase, no modification of dose in patients with renal insufficiency | Apnoea, muscular rigidity | Age< 1aa |
| Morphine | Age 1-5 months:  100mcg/Kg in ≥ 5 min  Age 6 months-11 aa:  100mcg/Kg in ≥ 5 min  Age ≥12 years:  5mg in ≥ 5 min | Age 1-5 months  10-30 mcg/Kg /h  Age 6 months-11 years:  20-30 mcg/Kg/h  Age ≥12aa:  20-30 mcg/Kg/h | 15-20 | 360-480 | Liver glucuronidation  Active metabolites | Respiratory depression, hypotension, urinary retention, biliary ductus spasm |  |
| Sufentanil* | 0,5-5 mcg/Kg | 0,05-4 mcg/Kg/h | Rapid | 50 | Liver N-dealkylation, O-dealkylation (cytochrome P450-3A4) and demethylation,  Intestinal metabolism  Inactive metabolites | Respiratory depression, apnoea, apnoea, muscular rigidity, bradycardia, hypotension | Age >1 month |
| Methadone | **Oral:** 100mcg/kg Dose may be increased up to effect, with steps of 50 mcg/Kg every 6 hours |  |  | Long duration, possible cumulative effects |  | Pheochromocytoma,  Q-T trait prolongation | Not authorized in pediatric age |
| ANTAGONIST | | | | | | | |
| Naloxone for opioids overdose | Age 1 month-11 years:  100 mcg/Kg repeatable 1 min later  (max 2 mg)  Age ≥12 years:  400 mcg then 800 mcg 1 min later, then 2 mg  (max 4 mg) | 60% of useful bolus dose/hour  Useful dose = dose which permits 15 min of spontaneous ventilation |  | Rapid | Liver glucuronidation | Arrhythmia, hypotension, hypertension, pulmonary oedema, cardiac arrest |  |
| Naloxone for  per respiratory and neurologic depression | Age 1 month-11 years:  1 mcg/Kg repeatable every 2-3 min  Age ≥12 aa:  100-200 mcg or 1,5-3 mcg/Kg then 100mcg every 2 min |  |  | Rapid | Liver glucuronidation | Arrhythmia, hypotension, hypertension, pulmonary oedema, cardiac arrest |  |

From BNFc 2020; *other sources

1. **Sedatives**

| **Molecule** | **Bolus dose** | **Continuous infusion dose** | **ONSET (min)** | **OFFSET (min)** | **Metabolism** | **Main side effects** | **OFF LABEL** |
| --- | --- | --- | --- | --- | --- | --- | --- |
| Dexmedetomidine* | 0,5-1 mcg/Kg  Slow bolus >10 min | 0,2-1,4 mcg/Kg/h | 15-20 | 120-180 | Liver N-glucuronidation N-methylation, oxidation  Minimally active metabolites | Bradycardia, atrioventricular block, hypotension, hypertension, hyperglycemia, hypoglycemia | Age > 18 years  Law 648/96  (GU 1674/2015) |
| Clonidine* | 3 mcg/Kg  Slow bolus | 0,3-2 mcg/Kg/h |  |  | Liver  Inactive metabolites | Bradycardia, hypotension, hypertensive rebound | Age > 18 aa |
| Midazolam | Age 6 mesi-11 years:  50-200 mcg/Kg in 3 min  Age ≥12 years:  30-300  mcg/Kg increasing with 1-2,5 mg step every 2 min | Age 1-5 months:  60 mcg/Kg/h  Age 6 months-11 years:  30-120 mcg/Kg/h  Age ≥12 years:  30-200 mcg/Kg/h | Rapid | 30-60 | Liver: hydroxylation (cytochrome P450-3A4)  Active metabolite 1-OH midazolam | Hypoventilation, apnoea, bradycardia, angioedema, paradox effect | See law 648/96 |
| Delorazepam* | **Oral:**  Age 1 month-11 years:  30-100 mcg/Kg  (max 4 mg)  Age ≥12 aa:  1-4 mg |  | 45 | Slow | Liver: N-glucuronidation  Inactive metabolites | Respiratory depression, paradox effect | Age < 5 years  See law 648/96 |
| Ketamine | Neonate: 0,5-2 mg/Kg  in 1 min  Age: 1 month-11 years  1-2 mg/kg  in 1 min  Age >12 years  1-4,5 mg/Kg  in 1 min | Neonates:  8-10 mcg/Kg/min  Children:  10-45 mcg/Kg/min | Rapid 30 sec | 5-10 | Liver: N-demethylation  Inactive metabolites | Sialorrhea, delirium, hallucinations, hypertonia | See law 648/96 |
| Propofol | Age 1 month-16 years:  2,5-4 mg/Kg  Age 17 years:  1,5-2,5 mg/Kg (20-40mg every 10 sec) | Age: 16-17 years  0,3-4 mg/Kg/h |  |  | Liver and lung conjugation | Anaphylaxis: angioedema, bronchospasm, hypotension, metabolic acidosis, hyperkalemia,  rhabdomyolysis, bradycardia, apnoea | Not authorized in continuous infusion in pediatric age |
| Sevoflurane* | AnaConDa® | **Delivery**  1,5-16,7 mL/h  Sevoflurane ET 0,6- 1,6 % | Rapid | Rapid | High pulmonary removal  Liver metabolism: defluoration and glucuronide conjugation  Production of inorganic fluoride | Liver and renal toxicity, hypotension, arrhythmias, bronchial hyperactivity, seizure | See law 648/96 |
| Sodium Thiopental | Neonates:  2 mg/Kg  Children:  4 mg/Kg | 1-5 mg/Kg/h* | 30-45 sec |  | Liver and partially kidney and brain | Arrhythmias, reduction of cardiac contractility, hypotension | See law 648/96 |
| Levomepromazine |  | Age 1 month-  11 years:  100-400 mcg/Kg/die  Age > 12 years:  5-25 mg/die |  |  | Liver sulfoxidation Demethylated active metabolite | Cardiac arrest, arrhythmias, liver toxicity, hyponatremia, hyperglycemia, delirium |  |
| \| ANTAGONIST \| \| --- \| | | | | | | | |
| Flumazenil | Neonate:  10 mcg/Kg every minute in 15 sec  Bambino:  10 mcg/Kg every minute  (max 200 mcg) in 15 sec  (max 2 mg; max 50 mcg/Kg each episode) | Neonates:  2-10 mcg/Kg/h  Children:  2-10 mcg/Kg/h  (max 400 mcg/h) | Rapid | 7-15 | Liver  Inactive metabolite | Anxiety, tachycardia, hypotension, hyperventilation tachycardia | Age < 1aa  Not authorized intravenous infusion |

From BNFc 2020; * other sources

1. **Neuromuscular Blocking Agents**

| **Molecule** | **Bolus dose** | **Continuous infusion dose** | **ONSET (min)** | **OFFSET (min)** | **Metabolism** | **Main side effects** | **OFF LABEL** |
| --- | --- | --- | --- | --- | --- | --- | --- |
| DEPOLARIZING |  |  |  |  |  |  |  |
| Succinylcholine* | 2 mg/kg | // | 1 | 5-10 | Plasmatic cholinesterase | Bradycardia, malignant hyperthermia, hyperkalemia |  |
| NON DEPOLARIZING |  |  |  |  |  |  |  |
| Atracurium | 300-600 mcg/Kg | 270-1770 mcg/Kg/h  Usual dose: 650-780 mcg/Kg/h | 3-4 | 20-35 | Hoffmann reaction | Seizure, hypotension | Age < 1 month |
| Cisatracurium | Age 1 month-1 years: 150 mcg/Kg  Age 2-11 years:  150 mcg/Kg  Age 12-17 years:  150 mcg/Kg | Age 2-11 years:  180 mcg/Kg/h then reducing to 60-120 mcg/Kg/h  Age 12-17 years:  180 mcg/Kg/h then reducing to 60-120 mcg/Kg/h | 2-3 | 30-60 | Hoffmann reaction | Bronchospasm, bradycardia | Age < 1 month |
| Vecuronium* | 100 mcg/kg | 0.5-10 mcg/Kg/min | 3-4 | 20-45 | Liver hydrolysis  Active metabolites with renal elimination | Hypotension |  |
| Rocuronium | 600 mcg/Kg | 300-600 mcg/Kg/h | 1-2 | 20-35 | Liver hydrolysis | Burning during infusion, hypotension |  |
| ANTAGONISTS |  |  |  |  |  |  |  |
| Neostigmine | Age 1 month-11 years:  50 mcg/Kg (max 2,5 mg)  Age 12-17 years:  50 mcg/Kg (max 2,5 mg)  in 1 min |  | 1-2 | 50-90 | Liver carbamate hydrolysis  Renal elimination | Bradycardia (it is necessary to use an anticholinergic drug) | Age < 1 month |
| Sugammadex* | Age 2-17 years:  2-16 mg/Kg |  | 2-4 | 60-120 | Renal elimination | Cutaneous rush, Q-T trait prolongation | Age < 2 years (to be use only to antagonize rocuronium) |

From BNFc 2020; * other sources

1. **Antipsychotics**

| **Molecule** | **Oral Bolus dose** | **Main side effects** | **Note** | **OFF LABEL** |
| --- | --- | --- | --- | --- |
| Haloperidol | Age 6-11 years: 0,25-1,5 mg Q 12h, 0,16-1 mg Q 8h  Age 12-17 years: 0,25-2,5 mg Q 12h, 0,16-1,6 mg Q 8h | Extrapyramidal signs, hyperglycemia, malignant neuroleptic syndrome, hyperlipemia, hematic dyscrasia, cutaneous rush | Possible subcutis route | < 3 years |
| Chlorpromazine | Age 1-5 years: 500mcg/Kg Q 4-6h (max 40 mg/die)  Age 6-11 years: 10 mg Q 8h (max 75 mg/die)  Age 12-17 years: 25 mg Q 8h, or 75 mg/die, possible increasing up to 300mg/die (max 1 g/die) | Extrapyramidal signs, hyperglycemia, malignant neuroleptic syndrome, hyperlipemia, hematic dyscrasia, cutaneous rush | Possible intravenous and intramuscular route | < 6 months |
| Risperidone | Age 5-17 years (body weight 15-20Kg): 250mcg for the first 4 dies, then 500mcg, finally increasing 250 mcg every day (max 1 mg/die)  Age 5-17 years (body weight 20-45Kg): 500mcg for the first 4 dies, then 1 mg, finally increasing 500 mcg every day (max 2.5 mg/die)  Age 5-17 years (body weight >45Kg): 500mcg for the first 4 dies, then 1 mg, finally increasing 500 mcg every day (max 3 mg/die) | Body weight increasing, hepatotoxicity , seizure, extrapyramidal signs, hyperglycemia, malignant neuroleptic syndrome, hyperlipemia, hematic dyscrasia, cutaneous rush | Possible intramuscular route | < 5 years |
| Olanzapine | Age 12-17 years:  15 mg/die,  Usual dose 5-20 mg/die.  (max 20 mg/die) | Body weight increasing, hepatotoxicity , seizure, extrapyramidal signs, hyperglycemia, hyperlipemia, hematic dyscrasia, cutaneous rush | Possible intramuscular route | < 13 years  648/96 |
| Quetiapine | Age 12-17 years:  1° day: 25 mg Q 2h  2° day: 50 mg Q 2h  3° day: 100 mg Q 2h  4° day: 150 mg Q 2h  5° day: 200 mg Q 2h  If necessary, increasable 100 mg/die | Body weight increasing, hepatotoxicity , extrapyramidal signs, hyperglycemia, hyperlipemia, cutaneous rush | // | <10 years  See law 648/96 |
| Aripiprazole | Age 13-17 years:  2 mg/die x the first 2 days, then 5 mg for the 3° and 4° days, finally 10 mg for the 5° and 6° days. If necessary, increasable 5 mg/die (max 30 mg/die) | Body weight increasing, hepatotoxicity , extrapyramidal signs, hyperglycemia, hyperlipemia, hematic dyscrasia, cutaneous rush | Possible intramuscular route | < 6 years |

From BNFc 2020
