# Supplementary material for: Recommendations for analgesia and sedation in critically ill children admitted to intensive care unit
Source: J Anesth Analg Crit Care. 2022 Feb 12;2:9. doi: 10.1186/s44158-022-00036-9 (PMC8853329; doi:10.1186/s44158-022-00036-9)
Supplement: Supplementary file 6 — Additional file 6. Drug interactions (file: Suppl mat 6). [file 44158_2022_36_MOESM6_ESM.docx]

**DRUG INTERACTIONS**

Concomitant administration of two or more drugs may induce a therapeutic effect or a drug interaction. Drug interactions may be physical-chemical, pharmacokinetic or pharmacodynamic. An adverse reaction with toxicity or therapeutic inefficacy may appear.

Clinicians should evaluate all possible drug interactions when prescribing therapy. Unfortunately, at present, in literature, knowledge of interactions and their effects on patients is not complete. A list of validated drug interactions is lacking. Studies on possible interactions of drugs before placing them on the market are difficult to perform due to the impossibility to evaluate their effects in vivo.

The aim of this Section is to report the known clinically relevant interactions of drugs presented in these Guidelines.

The reported list is not complete but may give clinicians directions with the help of pharmacologists and further considering literature.

**Note:**

**All analgesic and sedative molecules (not neuromuscular blocking agents) listed below may interact with each other causing additive effects on Central Nervous System**

*Opiates, benzodiazepines, ketamine, propofol, barbiturates, neuroleptics, antihistamines, antiepileptics, gabapentin, dantrolene*

**Mechanism**: additive effect on Central Nervous System and Respiratory System **Effects**: increased risk of respiratory depression/excessive sedation

1. **OPIATES**

**(morphine, fentanyl, remifentanil, sufentanil, methadone)**

- **Antagonists**

*Naloxone*

**Mechanism**: competitive binding to Receptors **Effects**: opiates withdrawal syndrome

- **Molecules causing inhibition of CYP3A4**

*Antimycotics azole, macrolides, antiretrovirals, protease inhibitors, phenobarbital, verapamil*

**Mechanism**: inhibition of metabolism (mediated by cytochrome P450 3A4) **Effects**: increased bioavailability of opiates with risk of toxicity (respiratory depression/excessive sedation)

- **Molecules causing induction of CYP3A4**

*carbamazepine, phenytoin, phenobarbital, rifampicin, tipranavir*

**Mechanism**: induction of metabolism of opiates (mediated by P450 3A4) **Effects**: decreased bioavailability with risk of withdrawal syndrome

- **Molecules causing the serotoninergic syndrome**

*Antidepressant selective inhibitors of serotonin reuptake, tricyclic antidepressant inhibitors of serotonin and norepinephrine*

**Mechanism**: unknown/additive effects with excessive serotoninergic stimulation **Effects**: increased risk of the serotoninergic syndrome

- **Molecules causing an additive effect on Central Nervous System**

*Antidepressants non-selective inhibitors of monoaminoxidase, isoniazid; linezolid*

**Mechanism**: additive depression on Central Nervous System; rare excitatory reactions are probably due to excessive serotoninergic activity in Central Nervous System (i.e. serotoninergic syndrome) **Effects**: depression of Central Nervous System; rare excitatory reactions (agitation, headache, diaphoresis, hyperpyrexia, rigidity, hypertensive crisis, hypotension, seizure, coma)

PARTICULARITY

**MORPHINE**

- **Molecules which elimination is decreased due to morphine**

*esmolol*

**Mechanism**: reduction of clearance (increased plasmatic level) **Effects**: increased adverse effects

- **Molecules with pharmacokinetic interaction with morphine**

*delta-9-tetraidrocannabinole*

**Mechanism:** delayed absorption of morphine, probably due to slow gastrointestinal mobility induced by cannabinoids (including delta-9-tetraidrocannabinile, a major component of Cannabis sativa) **Effects:** reduction of morphine effects

- **Molecules inhibiting GLICOPROTEINE-P**

*verapamil*

**Mechanism:** inhibition of P-glycoprotein, transmembrane carrier involved in absorption, distribution and/or elimination of methadone **Effects:** increased effect of methadone

**FENTANIL**

- **Molecules with antiseizure effect**

*tapentadol, tramadol*

**Mechanism**: additive effect **Effects**: increased risk of seizure

**METHADONE**

- **Molecules causing additive effect on QT trait prolongation**

*Inotropes, beta blockings, antiarrhythmics, macrolides, atypical antipsychotics, antimycotics azole, fluoroquinolones, furosemide, piperacillin, chloroquine, domperidone, ondansetron, tramadol, methadone, metronidazole, salbutamol, sevoflurane*

**Mechanism**: additive effect on QT trait prolongation **Effects**: increased risk of cardiotoxicity (torsade de pointes, cardiac arrest)

- **Molecules which metabolism is inhibited by methadone**

*dextromethorphan*

**Mechanism:** inhibition of CYP2D6 due to methadone **Effects:** possible increase of adverse effects of dextromethorphane

1. **BENZODIAZEPINES**

**(midazolam, delorazepam)**

- **Molecules causing inhibition of CYP3A4:**

*Antimycotics azole, macrolides, antiretrovirals, protease inhibitors, phenobarbital, verapamil*

**Mechanism**: inhibition of metabolism mediated by cytochrome P4503A4. E**ffects**: increased level of benzodiazepine and risk of adverse effects

- **Molecules antagonist of the antiseizure effect**

*flumazenil*

**Mechanism**: antagonism of antiseizure effect of benzodiazepines **Effects**: may promote seizure in patients treated with benzodiazepine or in patients physically addicted to benzodiazepine

PARTICULARITY

**MIDAZOLAM**

- **Molecules competing with enzymes involved in the metabolism of benzodiazepine**

*phenytoin*

**Mechanism**: enzymatic competition **Effects**: increased plasmatic level of one of the two molecules

**DELORAZEPAM**

- **Molecules with effect on the metabolism of benzodiazepine**

*Phenobarbital, carbamazepine*

**Mechanism**: possible induction of metabolism of delorazepam (CYP3A4) **Effects**: possible decreased plasmatic level of delorazepam with a possible decrease of its efficacy

1. **ALPHA AGONISTS**

**(clonidine, dexmedetomidine)**

**CLONIDINE**

- **Molecules active on alpha -adrenergic receptors**

*Beta-blockers*

**Mechanism**: increased response of alpha-adrenergic receptors **Effects**: rebound effect (hypertensive crisis) after stopping clonidine

- **Molecules with antagonism on alpha 2-adrenergic receptors**

*Tricyclic antidepressant*

**Mechanism**: antagonism on alpha 2-adrenergic receptors **Effects**: decreased antihypertensive effect

- **Molecules with cardiac effects**

*Calcio antagonists*

**Mechanism**: unknown **Effects**: increased risk of sinus bradycardia; rebound effect (hypertensive crisis) after stopping clonidine

- **Molecules with effects on Immune-System**

*ciclosporin*

**Mechanism:** unknown **Effects**: increased risk of toxicity of ciclosporin

- **Anaesthetic drug**

*mepivacaine*

**Mechanism:** unknown **Effects**: prolonged motor and sensitive block

**DEXMEDETOMIDINE**

*tacrolimus*

**Mechanism:** inhibition of tacrolimus (mediated by cytochrome P450 3A4) **Effects:** increased plasmatic level and risk of toxicity of tacrolimus (nephrotoxicity, hyperglycemia, hyperkalemia)

1. **ADJUVANTS**

**(ketamine, propofol, thiopental, sevoflurane)**

**KETAMINE**

- **Molecules causing cardiovascular depression**

*enflurane*

**Mechanism**: unknown **Effects**: cardiovascular depression

- **Molecules with effect on systemic pressure**

*Monoaminoxidase inhibitors, linezolid*

**Mechanism**: unknown **Effects**: hypotensive or hypertensive crisis

- **Molecules causing hypertension**

*pancuronium*

**Mechanism**: unknown **Effects**: hypotension

- **Molecules with pharmacokinetics interactions**

*diazepam*

**Mechanism:** reduction of clearance of ketamine, with increased plasmatic levels **Effects:** prolonged effect

- **Molecules with other interactions**

*theophylline, aminophylline*

**Mechanism:** unknown **Effects:** increased risk of seizure

**PROPOFOL**

- **Molecules with hypnotic effects**

*lidocaine*

**Mechanism**: unknown **Effects**: increased hypnotic effect

**THIOPENTAL**

- **Molecules with increased metabolism**

*aminophylline, theophylline, quetiapine*

**Mechanism**: induction of hepatic metabolism **Effects**: reduction of plasmatic levels

- **Molecules causing increased histaminergic response**

*succinylcholine*

**Mechanism**: increased histaminergic response **Effects**: intravascular disseminated coagulation

- **Molecules with metabolism inducted by thiopental**

*Steroids, sevoflurane, paracetamol*

**Mechanism:** induction of hepatic metabolism **Effects**: decreased effect of the administered drug

**SEVOFLURANE**

- **Molecules causing an additive effect on QT trait prolongation**

*Inotropes, beta blockings, antiarrhythmics, macrolides, atypical antipsychotics, antimycotics azole, fluoroquinolones, furosemide, piperacillin, chloroquine, domperidone, ondansetron, tramadol, methadone, metronidazole, salbutamol*

**Mechanism**: additive effect on QT trait prolongation **Effects**: increased risk of cardiotoxicity (torsade de pointes, cardiac arrest)

1. **NEUROMUSCULAR BLOCKING AGENTS (atracurium, cisatracurium, rocuronium, succinylcholine) AND ANTIDOTE (sugammadex)**

- **Molecules with additive action**

*Aminoglycosides*

**Mechanism**: additive or synergic neuromuscular block **Effects**: increased or prolonged neuromuscular block with respiratory depression or paralysis

PARTICULARITY

**ROCURONIUM**

- **Molecules decreasing efficacy of rocuronium**

*Steroids*

**Mechanism:** unknown **Effects:** reduction of efficacy of rocuronium, prolonged weakness and myopathy

- **Molecules causing induction of CYP3A4**

*carbamazepine*

**Mechanism:** induction of metabolism of rocuronium (mediated by cytochrome P450 3A4) because carbamazepine is a potent inductor of 3A4 **Effects:** reduced duration of neuromuscular block

**SUCCINYLCHOLINE**

- **Molecules causing increased efficacy of succinylcholine**

*Aminoglycosides, thiopental, benzodiazepines, lidocaine*

**Mechanism:** unknown **Effects:** increased depression of Central Nervous System and increased duration of neuromuscular block

***ANTIDOTE: SUGAMMADEX***

- **Molecules with anticoagulative action**

*heparin, warfarin*

**Mechanism**: pharmacodynamic interaction **Effects**: increased risk of bleeding (prolonged aPTT and PT)

1. **ANTIPSYCHOTICS**

**(Haloperidol, chlorpromazine, levomepromazine) and ATYPICAL NEUROLEPTICS or second-generation neuroleptics (olanzapine, risperidone)**

- **Molecules causing an additive effect on QT trait prolongation**

*Inotropes, beta blockings, antiarrhythmics, macrolides, antimycotics azole, fluoroquinolones, furosemide, piperacillin, chloroquine, domperidone, ondansetron, tramadol, methadone, metronidazole, salbutamol, sevoflurane*

**Mechanism**: additive effect on QT trait prolongation **Effects**: increased risk of cardiotoxicity (torsade de pointes, cardiac arrest)

PARTICULARITY:

**RISPERIDONE:**

- **Molecules competing for binding with plasmatic proteins**

*valproic acid*

**Mechanism**: possible shift of valproic acid **Effects**: possible increased of plasmatic levels of valproic acid and its toxicity

- **Molecules with metabolic interactions**

*phenytoin*

**Mechanism**: possible inhibition of phenytoin **Effects**: possible increased of plasmatic levels of valproic acid and its toxicity
